# The Irish DNA Atlas: Revealing Fine-Scale Population Structure and History within Ireland

**DOI:** 10.1038/s41598-017-17124-4

**Published:** 2017-12-08

**Authors:** Edmund Gilbert, Seamus O’Reilly, Michael Merrigan, Darren McGettigan, Anne M. Molloy, Lawrence C. Brody, Walter Bodmer, Katarzyna Hutnik, Sean Ennis, Daniel J. Lawson, James F. Wilson, Gianpiero L. Cavalleri

**Affiliations:** 1 Molecular and Cellular Therapeutics, Royal College of Surgeons in Ireland, St Stephen’s Green, Dublin 2, Ireland; 2 Genealogical Society of Ireland, Dún Laoghaire, Ireland; 3 School of Medicine, Trinity College, Dublin 2, Ireland; 4 Genome Technology Branch, National Human Genome Research Institute, National Institutes of Health, Bethesda, MD 20892 USA; 5 Weatherall Institute of Molecular Medicine and Department of Oncology, University of Oxford, Oxford, OX3 7DQ UK; 6 School of Medicine and Medical Science, University College Dublin, Dublin, Ireland; 7 University of Bristol, Department of Mathematics, University Walk, Bristol, BS8 1TW UK; 8 Centre for Global Health Research, Usher Institute for Population Health Sciences and Informatics, University of Edinburgh, Teviot Place, Edinburgh, Scotland; 9 MRC Human Genetics Unit, Institute of Genetics and Molecular Medicine, University of Edinburgh, Western General Hospital, Crewe Road, Edinburgh, Scotland; 10 The FutureNeuro Research Centre, Dublin, Ireland

## Abstract

The extent of population structure within Ireland is largely unknown, as is the impact of historical migrations. Here we illustrate fine-scale genetic structure across Ireland that follows geographic boundaries and present evidence of admixture events into Ireland. Utilising the ‘Irish DNA Atlas’, a cohort (n = 194) of Irish individuals with four generations of ancestry linked to specific regions in Ireland, in combination with 2,039 individuals from the Peoples of the British Isles dataset, we show that the Irish population can be divided in 10 distinct geographically stratified genetic clusters; seven of ‘Gaelic’ Irish ancestry, and three of shared Irish-British ancestry. In addition we observe a major genetic barrier to the north of Ireland in Ulster. Using a reference of 6,760 European individuals and two ancient Irish genomes, we demonstrate high levels of North-West French-like and West Norwegian-like ancestry within Ireland. We show that that our ‘Gaelic’ Irish clusters present homogenous levels of ancient Irish ancestries. We additionally detect admixture events that provide evidence of Norse-Viking gene flow into Ireland, and reflect the Ulster Plantations. Our work informs both on Irish history, as well as the study of Mendelian and complex disease genetics involving populations of Irish ancestry.

## Introduction

Located off the North-Western seaboard of Europe, Ireland’s geographic situation is conducive to genetic homogeneity and isolation. Indeed, several traits are found to be at high frequencies within the Irish, compared to the mainland European populations, including; cystic fibrosis^1^, lactase persistence^2^, coeliac disease^3^, galactosaemia^4,5^, and multiple sclerosis^6^. Studies of ancient Irish genomes suggest that the modern Irish genetic landscape was established about 3,500 years ago in the Irish Bronze Age^7^. There have since been a number of significant historical migrations into Ireland; the Norse Vikings in the late first millennium^8^, the Norman invasion of the 12^th^ century^9^, and the Plantations of the 16^th^ and 17^th^ centuries^9^. The impact of these migrations on the modern Irish genome is largely unknown.

Previous attempts to describe genetic structure in the Irish population primarily employed uniparental markers. Y-chromosome and mitochondrial haplotypes that are common within Ireland show genetic continuity with those observed in other western European populations^10,11^. Within the Irish population, there appears to be significant geographic structure in Y-chromosome haplotypes. There exists a general east-west cline of declining Y-chromosome haplotype diversity^10^, evidence of hegemony in the north-west of Ireland^12^, and in Munster, two haplotypes associated with the north and south of that province, respectively^13^. Furthermore, analysis of putatively Norse-origin surnames suggests little Norse Y-chromosome introgression within Ireland^14^.

The first autosomal evidence for population structure within Ireland resulted from analysis of ABO and Rhesus blood group frequencies^15–17^. Despite the limited resolution, this early work showed a general east-west cline in blood groups, mirroring the Y-chromosome results that would follow. Recent work with dense, genome-wide SNP data has established that Irish haplotype diversity is reduced relative to other European populations^18,19^, and both Runs of Homozygosity (ROH) and Linkage Disequilibrium (LD) have been found to be slightly elevated in Irish genomes^18,20^. Previous attempts have been unable to detect population structure within Ireland^20^, only observing that the population appears to be divergent from the neighbouring Scottish and English populations^18^. A limitation of these studies was a lack of geocoded data sampling, preventing any analysis of regions within these populations. Recent work has demonstrated fine-scale structure within the British Isles^21^. However the extent of such structure across the island of Ireland is unknown, and such a description would facilitate disease gene mapping within Ireland, as well as complementing similar efforts involving populations with Irish ancestry^21–23^.

In the current study we report on the assembly of a cohort of individuals with extended ancestry from specific regions in Ireland. Using dense genome-wide SNP genotype data we have performed a number of analyses to investigate population structure and extent of admixture within Ireland. We first utilize both fineStructure^24^ to investigate the extent of fine-scale population ‘clusters’, and Estimated Effective Migration Surfaces^25^ analysis to visualize regions of low or high genetic migration within Ireland and Britain. Second, we apply regression-based admixture modelling analysis^21^ and admixture event detecting software (Globetrotter^26^) to elucidate the extent of admixture in Ireland.

## Results

### Population Structure within Ireland

**Figure 1:**
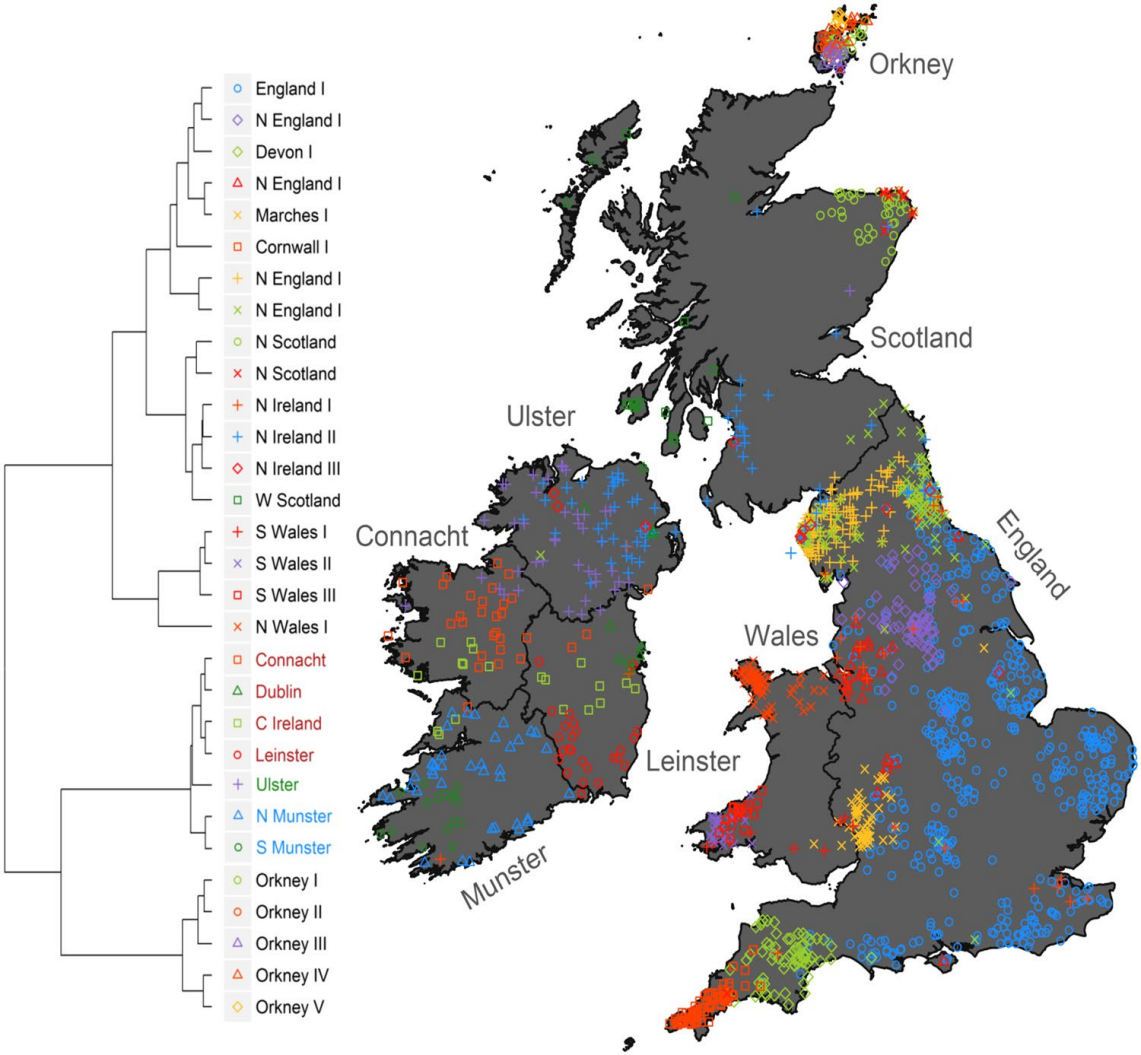
The clustering of individuals with Irish and British ancestry based solely on genetics. Shown are 30 clusters identified by fineStructure from 2,103 Irish and British individuals. The dendrogram (left) shows the tree of clusters inferred by fineStructure and the map (right) shows the geographic origin of 192 Atlas Irish individuals and 1,611 British individuals from the Peoples of the British Isles (PoBI) cohort, labelled according to fineStructure cluster membership. Individuals are placed at the average latitude and longitude of either their great-grandparental (Atlas) or grandparental (PoBI) birthplaces. Great Britain is separated into England, Scotland, and Wales. The island of Ireland is split into the four Provinces; Ulster, Connacht, Leinster, and Munster. The outline of Britain was sourced from Global Administrative Areas (2012). GADM database of Global Administrative Areas, version 2.0. www.gadm.org. The outline of Ireland was sourced from Open Street Map Ireland, Copyright OpenStreetMap Contributors, (https://www.openstreetmap.ie/) - data available under the Open Database Licence. The figure was plotted in the statistical software language R^46^, version 3.4.1, with various packages.

To investigate the extent of fine-scale population structure within Ireland, we assembled a combined SNP genotype dataset of 536 Irish (by which we mean henceforth Irish individuals from either the Irish DNA Atlas, Trinity, or PoBI datasets), 101 Scottish, 131 Welsh, 96 Orcadians and 1,239 English individuals. We performed fineStructure^24^ analysis on this dataset, which identified a final inferred state of k = 48 clusters. We then explored the hierarchal structure of the clustering, from the coarsest (*k* = 2) to the finest (*k* = 48) level. At *k* = 48 we reproduce previously reported genetic structure across Britain^21^, but in addition we observe seven large clusters of predominantly Irish membership (Supplementary Fig. 1), which we treat as putatively ‘Gaelic’ Irish. All seven of these ‘Gaelic’ Irish clusters are apparent by *k* = 30, and all clusters at this level have a minimum size of 10 individuals (Supplementary Table 1). At k = 30, we describe the finer Irish structure and also recapture previously described British structure^21^. In a similar fashion to previous reports, we present informative clusters with >10 individuals^21,27^ (see Fig. 1) so that downstream analyses had appropriate power. The results of the principal component analysis (PCA) of the fineStructure co-ancestry matrix are shown in Supplementary Data 3 and Supplementary Fig. 2. We also note the fineStructure approach outperformed Genome-wide Complex Trait Analysis (gcta64) (Supplementary Data 4 and Supplementary Fig. 3), observing that fineStructure is able to separate the ‘Gaelic’ Irish clusters at both coarse (Principal Components 1 and 2) and fine (Principal Components 7 and 8) levels better than gcta64.

The majority of our Irish individuals are placed in clusters grouped on one branch, which itself is grouped with the Orcadian branch. This combined branch forms an out group to the remaining fineStructure clusters (Fig. 1). The clusters found on the Irish branch are geographically stratified and we assigned labels to reflect this. To avoid confusion, a fineStructure cluster is referred to in italics (e.g. *Ulster*), distinct from the geographic region (i.e. the province of Ulster). Within the ‘Irish’ branch we observe three broad groups of clusters, which are indicated by the colour of the cluster’s label in the dendrogram shown in Fig. 1 cluster name colour in Fig. 1. The clusters describe; the north of Ireland (*Ulster*), the centre of Ireland (*Connacht*, *Dublin*, *C Ireland*, and *Leinster*), and the south of Ireland (*N Munster* and *S Munster*). The distribution of these Irish clusters follows both geographic and political borders within Ireland; particularly the boundaries of the four Irish provinces (see Fig. 1). The two clusters, *N Munster* and *S Munster*, follow the boundaries of Munster. The same can be said of the *Connacht* cluster for Connacht, and the *Ulster* cluster for Ulster. The centre of Ireland branch is predominantly found within the boundaries of the modern Irish province of Leinster, with the exception of the *C Ireland* cluster, which is also found within the north of Munster and the south of Connacht. In particular *Leinster* is found within the boundaries of the Leinster province and historical kingdom^28^. Finally, *Dublin* is mainly centred on the county of Dublin (with some members in the north in Ulster).

The fineStructure branch with the second largest proportion of Irish individuals is the North Ireland branch, containing the clusters *N Ireland I*, *II*, and *III* (Fig. 1A). These clusters are made up of Irish (predominantly from the north of Ireland), Scottish, and English (predominantly northern English) individuals to varying proportions. *N Ireland I* (n = 33) consists of 7 Irish and 26 English individuals, *N Ireland II* (n = 94) consists of 53 Irish, 19 Scottish, and 22 English individuals, and *N Ireland III* (n = 38) consists of 28 Irish, 1 Scottish, and 9 English individuals. The majority of the Irish individuals placed in a N Ireland cluster are found within *N Ireland II*, and their recent genealogical ancestry originates in Ulster (Fig. 1). The other Irish individuals in *N Ireland I* and *III* are predominately found within Ulster, or Dublin – though there is one individual in *N Ireland I* whose recent genealogical ancestry is from the south of Ireland. The mixed membership of these clusters suggests that these individuals have shared Irish and British ancestry. In order to further explore this hypothesis we utilised the genealogical data from the Irish DNA Atlas, comparing great-grandparental surnames from different clusters. We first classified surnames according to the following categories; Irish Gaelic, English (which included English or Anglo-Norman surnames), Scottish (which included Scottish or Gallowglass surnames), or other. We then compared whether Atlas individuals in the ‘Gaelic’ Irish and *N Ireland* clusters had significantly different surname counts of Irish or English, or Irish or Scottish origin. We tested using Fisher’s Exact Test in the computing language, R. We observe that the *N Ireland* clusters have both a significantly larger proportion of English surnames (*p* = 2.2e-16, OR: 6.34) and Scottish surnames (*p* = 2.2e-16, OR: 25.27) than the neighbouring ‘Gaelic’ Irish clusters.

To further compare the genetic distances between the observed Irish and British clusters, we performed F_st_ analysis, computing the F_st_ value between Irish and British fineStructure clusters (see Supplementary Table 2). As expected, and consistent with the fineStructure analyses and previous estimates^18,21^, genetic differentiation across Ireland and Britain is subtle, with the greatest genetic distances between Orcadian and non-Orcadian clusters (mean F_st_ = 0.0032). *Ulster* appears to be an outlier relative to the other ‘Gaelic’ Irish clusters, consistent with its position in PCA (Supplementary Fig. 2). The Gaelic clusters exhibit fine differentiation between each other (average F_st_ = 0.00030; average F_st_ excluding outlier *Ulster* = 0.00024) which is comparable to the differentiation we see between English clusters (average F_st_ = 0.00031; average F_st_ excluding outlier *Cornwall I* = 0.00024). This level of differentiation is finer than what we observe within Wales (average F_st_ = 0.00138), or Scotland (average F_st_ = 0.00250). The level of differentiation we observe in the island of Ireland (F_st_ = 0.0003), Gaelic and N Ireland clusters included, is almost an order of magnitude smaller than what we observe within clusters found across Great Britain, excluding Orkney (F_st_ = 0.00135).

Whilst the haplotypic relationship between two published ancient Irish genomes and modern European population has been described^7^, we were interested in whether we could detect any haplotypic affinity from these ancient Irish genomes to groups we observe within modern Ireland. We utilised a similar procedure to the original authors,^7^, performed ChromoPainter analysis and compared the haplotypic affinity of our modern Irish and British clusters to the Irish Neolithic Ballynahatty (3343–3020 cal BC) and Irish Bronze Age Rathlin1 (2026–1534 cal BC) individuals. We recorded the average length of haplotypic donation from each ancient Irish individual to each *k* = 30 Irish or British cluster (see Supplemental Data 5). We observe that the majority of clusters within Ireland and Britain share a similar affinity with Ballynahatty, with no significant differences between individual Irish clusters (Supplementary Fig. 4a). The highest haplotypic donations for Rathlin1 are to modern ‘Celtic-speaking’ populations, i.e. Ireland, Wales, and Scotland. Though no donations to ‘Irish’ clusters appear significantly different from each other, both *Connacht* and *Dublin* do show the highest affinity with Rathlin1 (Supplementary Fig. 4b). These results suggest a homogenous contribution of these two ancient genomes to contemporary genetic structure in Ireland.

### Estimated Effective Migration Surfaces

**Figure 2:**
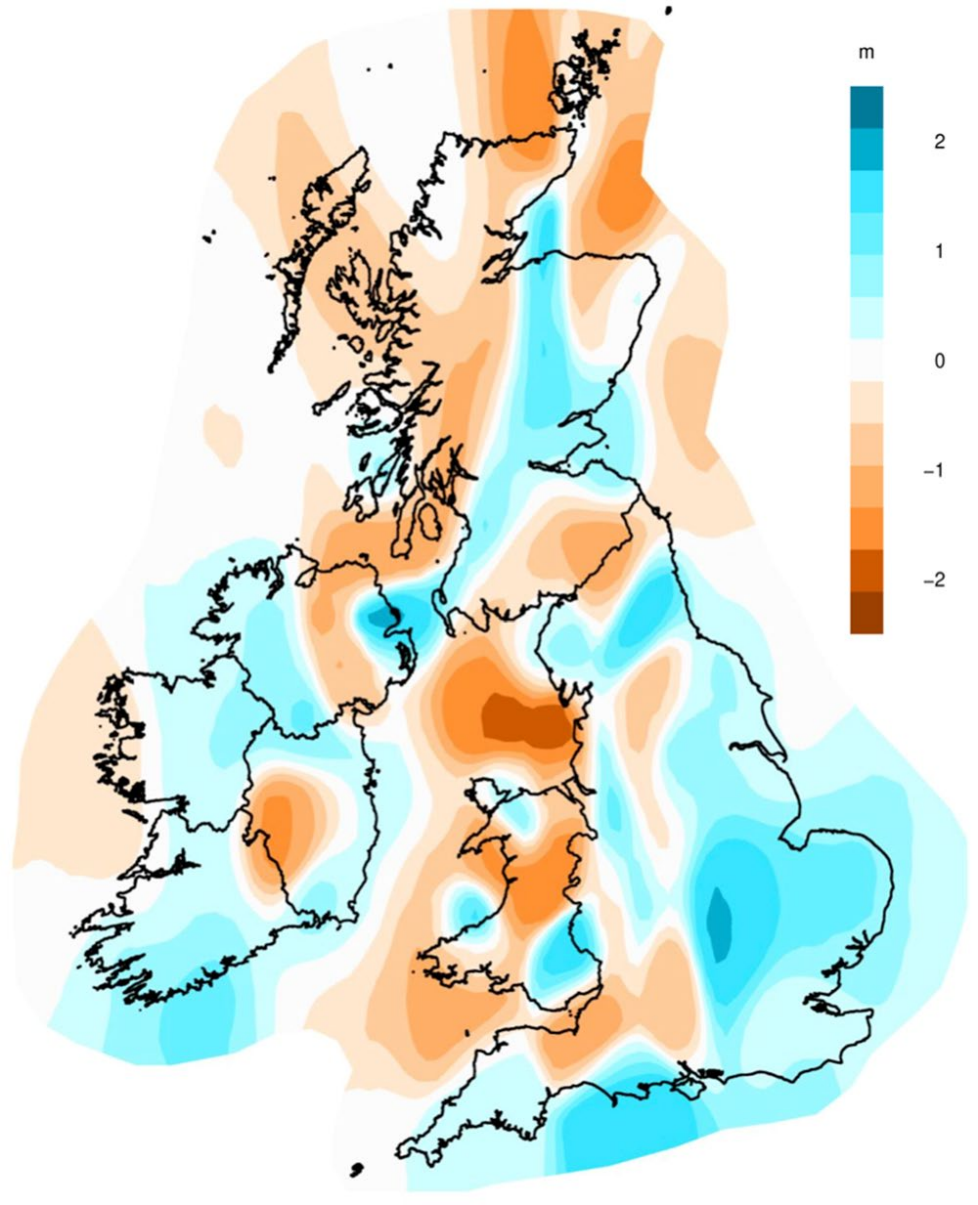
The estimated effective migration surface of Ireland and Britain from 1803 Irish and British individuals. Shown are the posterior mean migration rates of the six independent EEMS chains (*m* – on a log10 scale). The outline of Britain was sourced from Global Administrative Areas (2012). GADM database of Global Administrative Areas, version 2.0. www.gadm.org. The outline of Ireland was Open Street Map Ireland, Copyright OpenStreetMap Contributors, (https://www.openstreetmap.ie/) - data available under the Open Database Licence. The figure was produced in the statistical software language R^46^, version 3.4.1, with the package rEEMSplots.

In order to identify evidence of gene flow barriers within Ireland and Britain, we performed EEMS^25^ analysis on the Atlas and PoBI datasets. We did not include individuals from the Trinity dataset as they lack geocoding. For more details on the analysis see Methods and Supplementary Data 2.2. We observe a number of gene flow barriers within Ireland and the British Isles (Fig. 2). The strongest barrier is found around Wales between both Ireland and England, with a separate barrier observed between Scotland and Orkney. These barriers mirror the greatest divisions in our fineStructure and F_st_ analyses. We also observe several gene flow barriers within England. The first is in the south-west, and appears to separate out Devon/Cornwall from neighbouring English counties and Wales. The second is a region in the north of England – in the Pennine Hills – that is associated with the distribution of the fineStructure cluster *N England I*, with the third boundary following with the English-Scottish border. In addition to the general region of gene flow identified in south and central England, two notable corridors of gene flow are observed; the first runs along the Welsh-English border which represents the two clusters *N England II* and *Marches I*, and the second is in the north of England and represents the link between the two clusters *N England III* and *N England IV*. Within Scotland, a corridor of gene flow connects the two sampled regions, and there are two regions of gene flow in Wales that correspond to the areas where the majority of the north and south Welsh samples derive their ancestry from. The Western Isles and Highlands of Scotland present a large region of low gene flow, and could represent the relative isolation of the small number of samples from that region, which belong to the *W Scotland I* cluster.

In Ireland we detect a general trend of gene flow across the island, with three areas of low migration. The first is to the west of the island, including the coast of Connacht. The second is a region of relatively low genetic migration near the Leinster – Munster border. The final region of low genetic migration is found within Ulster, extending into Scotland, and seems to reflect the genetic differentiation of ‘Gaelic’ Ireland and Britain, specifically Scotland. This pattern of Ireland’s isolation is also seen in the gene flow barrier between Wales and Ireland. Interestingly we observe a corridor of relatively high genetic migration between the north-east of Ulster and the south-west of Scotland. This corridor appears to reflect the link between individuals of shared Irish and British ancestry (i.e. *N Ireland I*, *II*, and *III*), and indeed the authors of EEMS expect that under some circumstances EEMS could represent recent genetic migrants this way^25^.

### Ancestry Profiles

To explore the origins of the Irish genetic clusters we used a previously described method^21^ to model our Irish and British clusters as a mixture of different populations within Europe. If different clusters within Ireland have experienced dissimilar admixture histories within their past (with respect to Europe) this regression-based admixture analysis will demonstrate this with distinct admixture profiles.

In our analysis we estimated the ancestry profiles of each Irish and British cluster at *k* = 30. Prior to this, we performed fineStructure analysis on 6,021 European individuals to describe population structure in the European sample and inferred 134 clusters (see Methods). We then investigated the various hierarchal levels of the European clustering to identify a value of *k*-clusters which summarised the main population structure and retained clusters that are large enough to ensure the ancestry profile method’s accuracy and power. In order for our results to be comparable to previous uses of this method^21^ we chose a similar number of reference clusters using the European individuals. In the end, we identified *k* = 56 to be a good representation, i.e. the clusters represent the main branches of the dendrogram, yet retain enough samples for the regression admixture analysis. We additionally removed all individuals from putatively recent admixed clusters as we were interested in the ancestral haplotype diversity in each region of Europe. More recent admixture between reference European clusters would distort our ability to resolve more ancient contributions. This left a final *k* value of 51 clusters and 5,804 European individuals for the ancestry regression analysis. For more detail on the European clustering see Supplementary Table 4. We performed ChromoPainter haplotype painting of the Irish and British individuals, using the 51 European clusters as donors, and also painted the European individuals with the 51 European clusters donating haplotypes. We then solved, by regression, the average proportion of the genome in each Irish and British cluster that is closest, ancestrally, to each of the European clusters.

**Figure 3:**
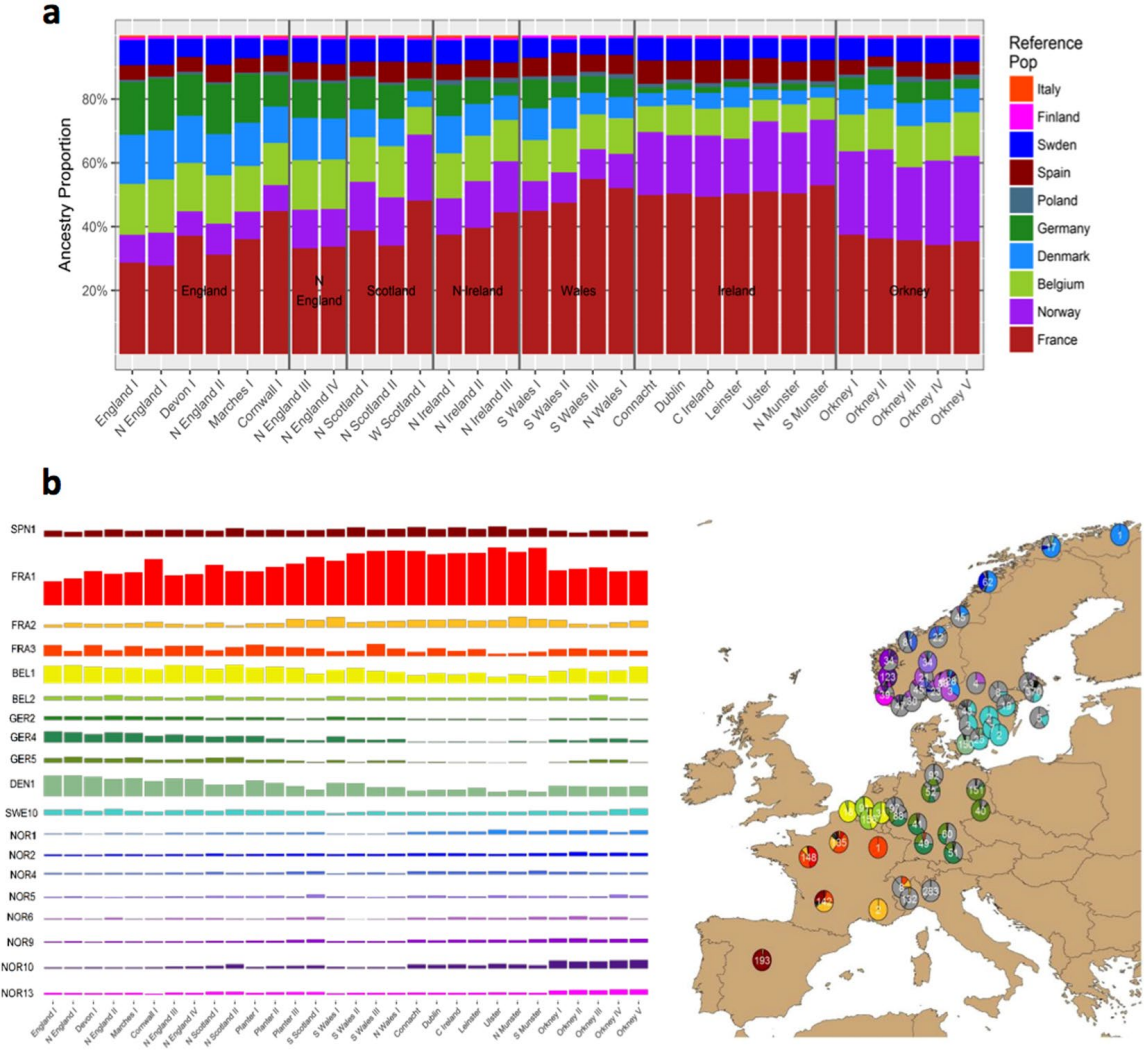
The European ancestry profiles of 30 Irish and British clusters. (**a**) The total ancestry contribution summarised by majority European country of origin to each of the 30 Irish and British clusters. (**b**) (left) The ancestry contributions of 19 European clusters that donate at least 2.5% ancestry to any one Irish or British cluster. (right) The geographic distribution of the 19 European clusters, shown as the proportion of individuals in each European region belonging to each of the 19 European clusters. The proportion of individuals form each European region not a member of the 19 European clusters is shown in grey. Total numbers of individuals from each region are shown in white text. Not all Europeans included in the analysis were phenotyped geographically. The figure was generated in the statistical software language R^46^, version 3.4.1, using various packages. The map of Europe was sourced from the R software package “mapdata” (https://CRAN.R-project.org/package=mapdata).

We report the total levels of ancestry proportions best represented by each group of European clusters grouped by broad country membership (Fig. 3a), and the ancestry proportions of the 19 individual European clusters that contribute at least 2.5% ancestry to any Irish or British cluster (Fig. 3b). The raw ancestry proportions are reported in Supplementary Table 5, and the 95% confidence intervals in Supplementary Table 6. For the seven ‘Gaelic’ Irish clusters, we observe that 80% of ancestry is best explained by clusters of French, Belgian, Danish, and Norwegian membership, with clusters from the other six reference European populations making up the remaining ~20% (Fig. 3a). French clusters are the best fit for about half of the ancestry within these Irish clusters, which is the highest proportion across all the Irish or British clusters. This French proportion is being driven primarily by the European cluster FRA1 which by itself represents an average of 30% ancestry in the ‘Gaelic’ Irish clusters (Fig. 3b). Cluster FRA1 is predominantly (80.0%) made up of individuals from the north-west region of France, an area with genetic affinity to other, British, ‘Celtic’ populations^23^. This pattern of French ancestry continues in other Irish and British clusters associated with Celtic ancestry; specifically the N Ireland, Scottish, Orcadian, Welsh, and Cornish clusters. The ‘Gaelic’ Irish clusters show the lowest ancestry proportions of German clusters, which in turn are thought to reflect Germanic/Saxon influence^21^. Orkney shows the second-least ‘Germanic’ proportion, with English clusters showing the most. We also observe a low amount of Belgian-like ancestry within Ireland, compared to groups within Britain, further illustrating Ireland’s relative isolation from mainland Europe.

In comparison to the ‘Gaelic’ Irish clusters, the N Ireland clusters show ancestry proportions that are variously in-between the Irish and the British proportions. Namely, as the proportion of German-like ancestry decreases, the proportion of France-like ancestry increases (r^2^ = 0.97, p = 0.08). This agrees with the proportions of individuals with recent Irish or British genealogical ancestry in each of the N Ireland clusters (see Results: Population Structure within Ireland), where the clusters with the least number of Irish individuals show the lowest proportion of French-like ancestry. Therefore, the N Ireland clusters appear to have arisen from different proportions of Irish and British ancestry.

A striking result of our admixture analysis is the surprising amount of Norwegian-like ancestry in our Irish clusters. We also detected high levels of Norwegian ancestry in Orcadian and Scottish clusters, and relatively low Norwegian ancestry in English and Welsh clusters. The Norwegian clusters that contribute significant ancestry to any Irish or British clusters predominantly consist of individuals from counties on the north or western coasts of Norway (Fig. 3b). These areas are noted to be regions where Norse Viking activity originated from^8^. Whilst this surprising Norwegian signal in Ireland is most likely due to Norwegian admixture into Ireland, indeed this would corroborate with accounts of Irish slave trade in the Viking era^29^, and Y-chromosomal analysis (unpublished). To test this hypothesis we ran an additional regression admixture analysis, this time modelling Norwegian haplotypes as a mixture of Irish, British, or European haplotypes (Supplementary Data 6). We observe significant proportions of Irish, Scottish, and Orcadian ancestry in modern Norway (6.82%, 2.29%, and 2.13%, respectively), particularly western Norway. This could provide evidence for Irish admixture back into Norway, but could also easily be explained by Norwegian haplotypes existing in Ireland, Scotland, and Orkney. Therefore, we are able to provide an upper estimate of ~20% Norwegian ancestry within Ireland, but unable to provide an empirical lower limit.

### Admixture within Ireland

In order to investigate evidence of admixture into Ireland from European sources we performed Globetrotter^26^ analysis on the combined European, Irish, and British dataset used in the regression-based ancestry profiling (see Methods). The Globetrotter method requires no *a priori* specification of admixture sources, instead modelling the source populations as a mixture of ‘surrogates’ who may or may not be ancestrally related to the actual source populations.

We analysed evidence of an admixture event in all individual ‘Gaelic’ Irish clusters, as well as in a combination of all the ‘Gaelic’ Irish clusters together (*Ireland-Combined*). We used the previously described 51 European clusters as surrogate source populations. We detected significant evidence of admixture (p < 0.01) in the *Ireland-Combined* cluster, as well as the; *C Ireland*, *Connacht*, *Leinster*, *N Munster*, and *Ulster* clusters (Supplementary Table 7). We observe dates of admixture events that collectively range from 38.72–29.92 generations (788CE–1052CE), involving two sources. The majority source is predominantly modelled as a mixture of FRA2 and FRA1 ancestry. The minority source contributed a range 0.31–0.36 ancestry with consistent French-like and German-like ancestry, and a northern European component, represented by the clusters DEN1, SWE10, and NOR1 and NOR10. These clusters contain individuals largely from regions associated with Viking activity. The majority source we interpret as the native Irish component, with the minor source the admixing source. The joint probability curves for the admixture analyses are presented in Supplementary Data 7 and Supplementary Fig. 7.1, and suggest the largest cluster tested, *Ireland-Combined*, has the cleanest signal for comparing major ‘Irish’ and ‘Norwegian’ components. Our Globetrotter results suggest an admixture event in Ireland involving a Scandinavian component that is dated to around the times of Viking activity in Ireland.

We hypothesised that clusters with both Irish and British membership (*N Ireland I*, *II*, and *III*), represent individuals with shared Irish and British ancestry due to admixture events. We therefore performed additional Globetrotter analyses, using the other 27 Irish and British clusters as surrogate source populations (see Methods). We found significant evidence of admixture in all three N Ireland clusters (p < 0.01) (see Supplementary Table 8), with dates ranging from the 17^th^ to the 18^th^ centuries. The largest of these clusters, *N Ireland II*, is estimated to have the oldest admixture date, 10.66 (CI: ±0.43) generations ago. We estimate this admixture event between two sources, contributing 0.34 and 0.66 ancestry. Source 1 is modelled as 0.52 native Irish ancestry (of which *C Ireland* accounts for 0.258, and *Ulster* – 0.260), with 0.8 of source 2 ancestry modelled as *N England IV*. The admixture events detected in the other two clusters, *N Ireland I*, and *N Ireland III*, have later dates (7.42 CI: ±0.43 and 9.24 CI: ±0.31, respectively). *N Ireland I* consists of source 1 contributing 0.4 and source 2 contributing 0.6 ancestry to the admixed population. Source 1 mainly consists of 0.298 *Leinster*, 0.189 *C Ireland*, and 0.152 *N England I* ancestry, with source 2 mainly consisting of 0.646 *England I* ancestry. Unlike *N Ireland I* and *II*, the major source of the *N Ireland III* admixture event is mainly Irish in ancestry. The major source 2 (0.7 total ancestry) consists mainly of *C Ireland* (0.412) and *Ulster* (0.162) ancestry. The minor source 1 (0.3 total ancestry) is modelled to consist mainly of English ancestry (*England I* – 0.332, *N England IV* – 0.322, *N England I* – 0.119, and *N England II* – 0.103). The joint probability curves of the majority ‘Irish’ and major ‘British’ components (Supplementary Fig. 7.2–4) show that the largest cluster (*N Ireland II*) demonstrates the cleanest signal. The fitted curves are relatively shallow compared to other admixture signals^21,26^, suggesting a relatively gradual admixture process.

## Discussion

We have investigated population structure and diversity within Ireland with high-density genome-wide SNP data of 192 individuals with four generations of Irish ancestry from specific regions around Ireland. Using haplotype based methods we have described remarkable fine-scale population structure within Ireland, complementing similar efforts in Britain^21^. We have also identified regions of Ireland and Britain that show differing levels of effective migration rates with low levels of gene flow across country borders. We have generated ancestry profiles, demonstrating that Ireland has a distinct profile compared to the British Isles, and evidence of widespread Norwegian-like ancestry in Ireland. Finally we have demonstrated evidence of admixture into Ireland, creating clusters of dual Irish and British membership, as well as a separate admixture event which involves a Scandinavian component introduced into Ireland.

Previous investigations into the extent of genetic structure within Ireland using autosomal^15–18,30^, or uniparental markers^10–14,31^, identified a general east-to-west cline in allele frequencies and evidence of population structure within both the north and the south of Ireland. Using fineStructure^24^ we identified seven clusters of predominantly Irish ancestry (‘Gaelic’ Irish), and three clusters of shared Irish and British ancestry (‘N Ireland’). The structure that we observe within our ‘Gaelic’ cluster in the north of Ireland (*Ulster*) and the two ‘Gaelic’ clusters in the southern Province of Munster (*N Munster* and *S Munster*) agree with earlier, Y-chromosome work^12,13^. The levels of differentiation between the ‘Gaelic’ clusters are small, comparable to what is found in Great Britain. This fine-scale structure is associated with evidence of few gene flow barriers within Ireland, which contrasts with a number of such barriers in Britain, which themselves are chiefly associated with ancient administrative boundaries. The fine nature of genetic structure in the ‘settled’ Irish also further emphasises the elevated levels of differentiation observed within Irish Travellers clusters (average F_st_ = 0.010)^32^, a population isolate found within Ireland.

As suggested by their nomenclature, the ‘Gaelic’ clusters we have identified are associated with geopolitical regions such as Provinces or historical kingdoms within Ireland. The degree to which the genetic clusters predict these historical groups is remarkable, compared to what is found within the majority of England, for example. These results suggest a lack of widespread movement in the recent history of Ireland, at least to up to the mid-19^th^ century. Additionally the clusters do not appear to have significantly different affinities to ancient Irish genomes, suggesting the structure we observe is not due to different proportions of ancient Irish ancestries. These clusters do however seem to reflect more recent historical events within Ireland. *N Munster* and *S* *Munster* together predict the boundaries of the province of Munster, and individually are associated with the boundaries of the kingdoms of Dál Cais and the Eóganacht^13,28^, respectively. Furthermore, within Munster, County Clare was originally under the rule of the people of Connacht, until being eventually acquired by Munster^28^. Our analysis mirrors this history, showing that individuals with recent ancestry from Co Clare are a mixture of genetic groups found both in Munster and in Connacht. In the north of Ireland, historically the people of Ulster (represented by the Uí Néill) and the people of Connacht (muinter Chonnact) have been linked – either by patrilineal ties or as military allies^28^. This is also mirrored in our clustering, where *Connacht* is found in areas associated with Ulster rule, and *Ulster* in areas of Connacht rule. The *Ulster* cluster itself shows the greatest genetic distance from Britain, in both our PCA and F_st_ analysis, despite its geographic proximity to Britain. Given that we have identified groups within the north of Ireland that do have genetic links to Britain, i.e. the N Ireland clusters, *Ulster* most likely represents individuals of ‘Gaelic’ ancestry that have remained genetically isolated from Britain – which reflects the demographic and political history of the region.

Our fine structure results agrees well with previous analyses of the PoBI dataset^21^, but adds a large sample of Irish so as to better understand the population structure of the region. The clustering and order of the dendrogram is largely the same, with the exception of Scotland and Ireland. We observe that the majority of our Irish individuals are found on one, ‘Gaelic’ Irish branch, which is grouped with the Orcadian branch. This grouping is likely due to the similar (but separate) genetic distances from Ireland and Orkney to the rest of the British populations, which fineStructure’s tree building algorithm interprets by branching together. Both Ireland and Orkney share elevated levels of Norwegian-related ancestry, which could provide an alternative explanation for this grouping. However our PCA suggests no large scale gene flow between the two populations. As well as observing groups in Ireland with links to Scotland, we observe the *W Scotland I* cluster to be closer to Ireland in our PCA than the other Scottish clusters, suggesting another link between south/west Scotland with north Ireland. Indeed this is the part of Scotland which has historically spoken Scots Gaelic. The PoBI dataset’s restricted Scottish coverage, which is largely northern Aberdeenshire and the south of Scotland, means we can only describe Scotland with regards to those regions. It would be interesting, therefore, to expand the Scottish sample further to investigate genetic links between Ireland and the rest of Scotland, particularly the Hebrides.

Considering the fine-scale nature of the population structure we observe within Ireland, the trend of gene flow across the island is unsurprising. Interestingly we observe a gene flow barrier within the province of Leinster. Speculating, this could represent the historical rivalry between Munster and Leinster^28^, or the split of Munster from the rest of the island. Co Clare’s shared Connacht and Munster history could have removed the Munster/Ireland barrier in that region. This would explain the region of low gene flow along the west coast of Ireland. Perhaps the most interesting feature of our analysis in Ireland is the genetic barrier between West Ulster/Ireland and East Ulster/Scotland, which represents the general divide within Irish genetics between the ‘Gaelic’ Irish clusters and the ‘N Ireland’ clusters. This is further supported by the reciprocal gene flow corridor between Scotland and East Ulster, presumably partly representing the geographic distribution of the largest ‘N Ireland’ cluster, *N Ireland II*. Within Britain we observe that the primary barriers to gene flow are along the ancient political boundaries between England, Wales, and Scotland, as well as between Scotland and Orkney. Additionally we observe a barrier separating Cornwall/Devon from the rest of England, and a strong barrier between north and south Wales. Whilst EEMS is less sensitive to sampling schemes than other methods of detecting genetic migration^25^, the authors do note that the migration surface is affected when there is limited sampling on one side of a genetic barrier, as is the case in our Scottish-English, and to a lesser degree Welsh-English comparisons. Therefore a more comprehensive sample in Wales and Scotland may further elucidate the migratory patterns in these regions.

In addition to the population structure found within Ireland, we have demonstrated that Ireland has a distinct ancestry profile in our regression-based admixture analysis. We identify a high level of France-like ancestry being driven by a single French cluster with high North-Western French membership. The North-West of France has previously been shown to have genetic links with Celtic populations in Britain^23^. Therefore the large signal we observe within Ireland could reflect Ireland as a ‘sink’ of Celtic ancestry, considering its isolation compared to other British Celtic groups. Considering the links from north-west France to other Celtic populations^23^, we do not interpret this as a ‘Norman’ signal. The ancestry profiles also consists of a surprising level of Norwegian related ancestry, especially considering previous attempts to detect ‘Norse Viking’ admixture into Ireland have been inconclusive^14^. Ireland presents the second highest amount of Norwegian ancestry in our analysis after Orkney, where Norse Viking admixture is a well-described feature^21,33,34^. All areas traditionally associated with Norse Viking activity (Ireland, Scotland, and Orkney) present relatively high levels of Norwegian-like ancestry. A ‘Norse Viking’ admixture event is further supported by our Globetrotter analysis, which detected significant admixture events into Ireland. This introduced a Norwegian/Scandinavian component corresponding with the time of historical Viking activity in Ireland. Furthermore, the Norwegian individuals that contribute this ancestry to Irish clusters are predominantly from areas in Norway where Norse Viking activity is known to originate from^8,28^. The effect of the Norse Vikings on the genetic landscape of Ireland seems to be shared across Ireland, and not limited to regions of Norse settlement, e.g. Limerick, Waterford, Wexford, and Dublin^8^. This suggests any structure that we are detecting in Ireland post-dates the introgression of this Norwegian-like ancestry. Alternatively the structure could be old and persistent, with a lack of strong genetic barriers resulting in a gradual homogenisation of Norse ancestry within modern Ireland. Whilst a proportion of the elevated Norwegian ancestry within Ireland could be due to Irish haplotypes in our modern Norwegian sample, our Globetrotter results suggest this is not the primary cause. This component could have been introduced by Vikings who were genetically Norwegian in ancestry, or individuals with mixed Scottish and Norwegian ancestry. If the latter is the case, this would explain our Globetrotter date estimates, which generally fall before the beginning of the Viking period in Ireland. Scottish haplotypes in the admixing source into Ireland would lower any admixture date estimate. This is likely, considering the low, but constant, level of gene flow expected across Ireland and Scotland prior to the Viking admixture. This hypothesis would also support the noisy and relatively shallow admixture signal we detect in the Globetrotter joint probability curves. A portion of this Norwegian-like ancestry could originate from a later, but smaller, admixture event due to Norman settlers into Ireland. However the Globetrotter date estimates disagree with this as the primary source of Norwegian admixture.

Finally, we have identified groups of people in Ireland who share recent genetic history with individuals in Britain. The three clusters (*N Ireland I*, *II*, and *III*) consist of Irish mainly from the north of Ireland, Scottish predominantly from the south of Scotland, and English mainly from the north of England. This dual ancestry is also reflected in the surnames of the Irish DNA Atlas individuals included in these clusters, who have an enrichment of both English and Scottish surnames compared to the ‘Gaelic’ Irish clusters. Their ancestry profiles show clusters that are midway between Ireland and Britain, which agrees with their intermediate position in our PCA. Our Globetrotter analysis of the clusters suggests they are a result of an admixture event between Irish and British dating between the 17^th^ and 18^th^ centuries. This coincides with the period of the Ulster Plantations, between the 16^th^ and 17^th^ centuries^9^, and our best fit for the source of the British component is a mixture of individuals from the centre/south of England (*England I*) and the north of England (*N England IV*). Whilst the EEMS analysis instead links the north of Ireland to south Scotland, this may be a result of the EEMS algorithm finding the best fit of the Ireland-Britain relationship by linking Ireland to Scotland, rather than to the more distant England. The Globetrotter joint probability curves suggest this admixture process was probably a gradual one and may be confounded by previous gene flow from Britain into Ireland (and *vice versa*). For example, the settling of the Scottish ‘galloglass’ mercenary dynasties from the late thirteenth to the early fifteenth centuries, or the hiring throughout the sixteenth century of very large numbers of Scottish ‘redshank’ mercenaries^35,36^, and ultimately historical low level migration between northern Ireland and northern Britain. The larger odds ratio we observe in our surname analysis for Scottish surnames rather than English surnames would also support the hypothesis of gradual Scottish gene flow.

In conclusion we have identified fine-scale genetic structure in Ireland that is geographically stratified and surprisingly faithful to the historical boundaries of Irish Provinces and kingdoms. We have detected two sources of admixture into Ireland. One is associated with a significant level of Norwegian ancestry, which we date to the time of Norse-Viking activity. The second is associated with Scottish and English ancestry and dates to the times of the Ulster Plantations. Our work informs on Irish history, and by outlining fine-scale structure across the island, we hope can aid the study of genetic diseases within Ireland, and others populations with Irish ancestry.

## Methods

### Study Populations

We assembled four datasets of individuals with European ancestry; the Irish DNA Atlas (n = 194), the Trinity Student Study dataset (n = 2232)^37^, the Peoples of the British Isles (PoBI) dataset (n = 2039)^38^, and the WTCCC2 Multiple Sclerosis dataset (n = 6,760)^39^.

The Irish DNA Atlas is a dataset of individuals with four generations ancestry within in Ireland, where great-grandparents are usually born with 50 km. Informed consent was obtained from all Irish DNA Atlas individuals, and the data collection and analysis of these individuals was carried out in accordance with the relevant guidelines and regulations approved by the Royal College of Surgeons in Ireland Research Committee, reference number REC0020563.

The Trinity Student dataset is a cohort of individuals with Irish ancestry. The PoBI dataset is a cohort of individuals with two generations of ancestry within 80 km within the United Kingdom. The Multiple Sclerosis dataset includes cases and controls of individuals from European centres, with the majority of European ancestry. For more information of each of the four datasets, see Supplemental Data 1.

### Quality Control of Genotype Data

Each of the four cohorts were individually processed through a number of quality control steps using the software PLINK 1.9^40,41^. Only autosomal SNPs were included. Individuals or SNPs that had >5% missing genotypes, SNPs with a minor allele frequency (MAF) <2%, and SNPs failing HWE at significance of <0.001 were discounted from further analysis.

Individuals included from the European ancestry dataset^39^ were genotyped as part of a study of multiple sclerosis (MS), which included cases. As the HLA region contains loci strongly associated with MS^39^, for any analyses that included the European individuals from this MS study we omitted SNPs from a 15 Mb region around the HLA gene region, starting at 22,915,594 to 37,945,593 (human genome build 38). In order to confine ourselves to European populations we also restricted our analyses to individuals from Belgium, Denmark, Finland, France, Germany, Italy, Norway, Poland, Spain, and Sweden. In order to remove significantly admixed individuals, we removed outlier individuals from the full dataset of these ten populations using smartpca from the eigensoft package^42^. We used the default values for outlier removal; using 5 iterations, 10 principal components, and removing individuals that exceeded 6 standard deviations from any of the 10 principal components. A further round of outlier elimination was carried out on each separate population using the same settings. This quality control left a final count of 6,021 MS European individuals.

For fineStructure analyses we restricted ourselves to markers shared between the four datasets after individual QC. This shared marker set included 256,379 markers in total. We also removed two individuals from the Atlas dataset as they shared a PIHAT score > 0.05 with another Atlas individual, as fineStructure is more sensitive to relatedness than other population structure detecting tools^24^.

### Population Structure within Ireland

We assembled a combined dataset of individuals with Irish and British ancestry, to detect population structure within Ireland, within the context of neighbouring Britain. We combined 192 Atlas Irish, 300 Trinity Irish, and 1611 PoBI British (which consisted of 1,239 English, 101 Scottish, 131 Welsh, 96 Orcadians, and 44 Northern Irish). We included additional Irish individuals from the Trinity Student dataset as we have found that increasing the sample size of a population improves the ability of fineStructure to be able to detect finer scale structure within that population. We used a subset of the PoBI dataset, reducing the numbers of English PoBI individuals, as we found this did not significantly change the clustering within Britain, and we were primarily interested in the structure detected within Ireland.

Briefly, we phased the combined dataset of 2,103 individuals and 256,379 common markers using SHAPEIT v2.r790^43^, using genetic map build 37. We converted the resultant haplotype files to ChromoPainter format and performed fineStructure^24^ analysis. For more details on the parameters used for the phasing and fineStructure analysis see Supplemental Data 2.1. We analysed the final inferred number of fineStructure *k* clusters and choose the number of clusters (*P*) which both best represented the final observed population structure and combined small uninformative clusters of >10 individuals. For more details of the fineStructure analysis see Supplemental Data 2.1.

In order to compare to more conventional methods of detecting population structure, we performed PCA using gcta64^44^. Using the combined dataset from the fineStructure analysis, we first pruned the dataset of SNPs with the plink command–indep-pairwise 1000 50 0.2. With a final dataset of 2,103 individuals and 79,417 common markers we created a genetic relationship matrix in gcta64, and performed PCA on it (generating the first 10 principal components).

In addition to PCA performed with gcta64, we also investigate population structure calculating F_st_ between the Irish and British fineStructure clusters using the Weir and Cockerham method^45^. This was calculated using the same individuals and common markers used in the Ireland and Britain fineStructure analysis.

### Estimated Effective Migration Surfaces

To further investigate the spatial patterns of genetic structure within Ireland and Britain we used the software Estimated Effective Migration Surfaces (EEMS)^25^, a method that visualises relative gene flow across a habitat using geo-coded genetic data. We used a subset of the combined dataset used in the Population Structure within Ireland analysis. We chose the 192 Atlas and 1611 PoBI individuals as they have latitudinal and longitudinal data. This final dataset included 1,803 individuals and 256,379 common SNPs. For more information on the specific EEMS pipeline we used, see Supplementary Data 2.2.

### Ancestry Profiles

In order to investigate haplotype diversity within Ireland compared to that found within the British Isles we performed a regression-based ‘ancestry profile’ method first described by Leslie *et al*.^21^. Using fineStructure ‘chunklength’ data (the amount, genome-wide in cM, that each fineStructure recipient individual copies from each donating source), we modelled each Irish and British fineStructure cluster identified in the Population Structure in Ireland analysis as a mixture of haplotypes that each most closely resemble the haplotype profile of different European reference fineStructure populations.

We first identified fineStructure clusters in a large dataset of individuals with European ancestry (the MS European dataset). We performed fineStructure analysis on the phased dataset of 6,021 individuals with the same 256,379 markers in the Population Structure in Ireland analysis, using default parameter values, with the exception of MCMC sampling iterations (1,000,000), MCMC burnin iterations (1,000,000), and tree building iterations (100,000). We analysed the final inferred tree and *k* clusters, and chose a value of *k*-clusters (*G*) to use in further analyses that represented the population structure observed within our European sample, but still retaining large enough fineStructure clusters for these analyses. We also removed clusters of recently admixed individuals.

We then performed ChromoPainter haplotype painting with fs2.1.0pre, painting our Irish and British individuals as copying haplotypes from European individuals and the final *G* European clusters and recorded the average amount of copying that each *P* cluster copies from each *G* cluster. We additionally painted each European individual as copying from every *G* European population (with individuals unable to copy from themselves).

Using the regression method, an adaptation of the non-negative-least-squares (nnls) function^41^ in R^46^, we estimated the proportion of ancestry each Irish and British cluster that most closely resembles the haplotype diversity found in each *G* European cluster. For more detail on the regression method, see Supplemental Data 2.3.

### Admixture within Ireland

In order to investigate evidence of admixture within Ireland we further analysed fineStructure clusters with shared Irish and British membership (*N Ireland I*, *II*, and *III*). This was performed on the same individuals and common markers that the Irish and British fineStructure analysis included, using the R^46^ program Globetrotter^26^. We investigate the evidence of each of the three clusters resulting from one or two admixture events between populations represented by the other Irish and British fineStructure clusters. We performed the Globetrotter analysis with the default values suggested in the manual. We generated the sample files (a record of which donating haplotype each target individual copies at each locus) by using ChromoPainterv2^24^ to conditionally paint the N Ireland haplotypes as copying haplotypes from every other Irish/British fineStructure cluster (with the exception of the other two N Ireland clusters). We generated a p-value for significance of detecting admixture with 120 bootstrap replicates, reporting the proportion of bootstraps with an inferred date of ≤1 or ≥400 generations as recommended by the authors^26^. We calculated the 95% confidence intervals of the inferred date with an additional 120 bootstrap replicates.

We also performed Globetrotter analysis investigating any admixture events into Ireland from mainland European sources. We used the combined dataset of 2,130 Irish and British, and 5,804 European individuals. We tested each individual ‘Irish’ cluster (*C Ireland*, *Connacht*, *Dublin*, *Leinster*, *S Munster*, *N Munster*, *Ulster*) and a combined population all ‘Irish’ clusters together (referred to as *Ireland-Combined*) for evidence of admixture using the 51 European fineStructure clusters as surrogates for any admixing source. We generated a p-value for significance of detecting admixture with 120 bootstrap replicates, reporting the proportion of bootstraps with an inferred date of ≤1 or ≥400 generations. We calculated the 95% confidence intervals of the inferred date with an additional 120 bootstrap replicates.

### Data Availability

For access to the Irish DNA Atlas genotype data, please contact the corresponding author. The other genotype data used in this study are previously published, please see the relevant study for the following datasets; Trinity Student Study dataset^37^, the Peoples of the British Isles dataset^38^, and the WTCCC2 Multiple Sclerosis dataset^39^.

## Electronic supplementary material

Supplemental Material

Supplemental Table 2

Supplemental Table 4

Supplemental Table 5

Supplemental Table 6

Supplemental Table 7

Supplemental Table 8
