# Author Correction: The Irish DNA Atlas: Revealing Fine-Scale Population Structure and History within Ireland

**DOI:** 10.1038/s41598-018-24846-6

**Published:** 2018-05-03

**Authors:** Edmund Gilbert, Seamus O’Reilly, Michael Merrigan, Darren McGettigan, Anne M. Molloy, Lawrence C. Brody, Walter Bodmer, Katarzyna Hutnik, Sean Ennis, Daniel J. Lawson, James F. Wilson, Gianpiero L. Cavalleri

**Affiliations:** 1 Molecular and Cellular Therapeutics, Royal College of Surgeons in Ireland, St Stephen’s Green, Dublin 2, Ireland; 2 Genealogical Society of Ireland, Dún Laoghaire, Ireland; 3 School of Medicine, Trinity College, Dublin 2, Ireland; 4 Genome Technology Branch, National Human Genome Research Institute, National Institutes of Health, Bethesda, MD 20892 USA; 5 Weatherall Institute of Molecular Medicine and Department of Oncology, University of Oxford, Oxford, OX3 7DQ UK; 6 School of Medicine and Medical Science, University College Dublin, Dublin, Ireland; 7 University of Bristol, Department of Mathematics, University Walk, Bristol, BS8 1TW UK; 8 Centre for Global Health Research, Usher Institute for Population Health Sciences and Informatics, University of Edinburgh, Teviot Place, Edinburgh, Scotland; 9 MRC Human Genetics Unit, Institute of Genetics and Molecular Medicine, University of Edinburgh, Western General Hospital, Crewe Road, Edinburgh, Scotland; 10 The FutureNeuro Research Centre, Dublin, Ireland

Correction to: *Scientific Reports* 10.1038/s41598-017-17124-4, published online 08 December 2017

This Article contains a low resolution version of Figure 3 and labels that are inconsistent with the main body text where the labels ‘Planter 1-3’ in panel b should read ‘N Ireland 1-3’. A higher resolution Figure 3 containing the updated figure labels appears below as Figure 1.

**Figure 1:**
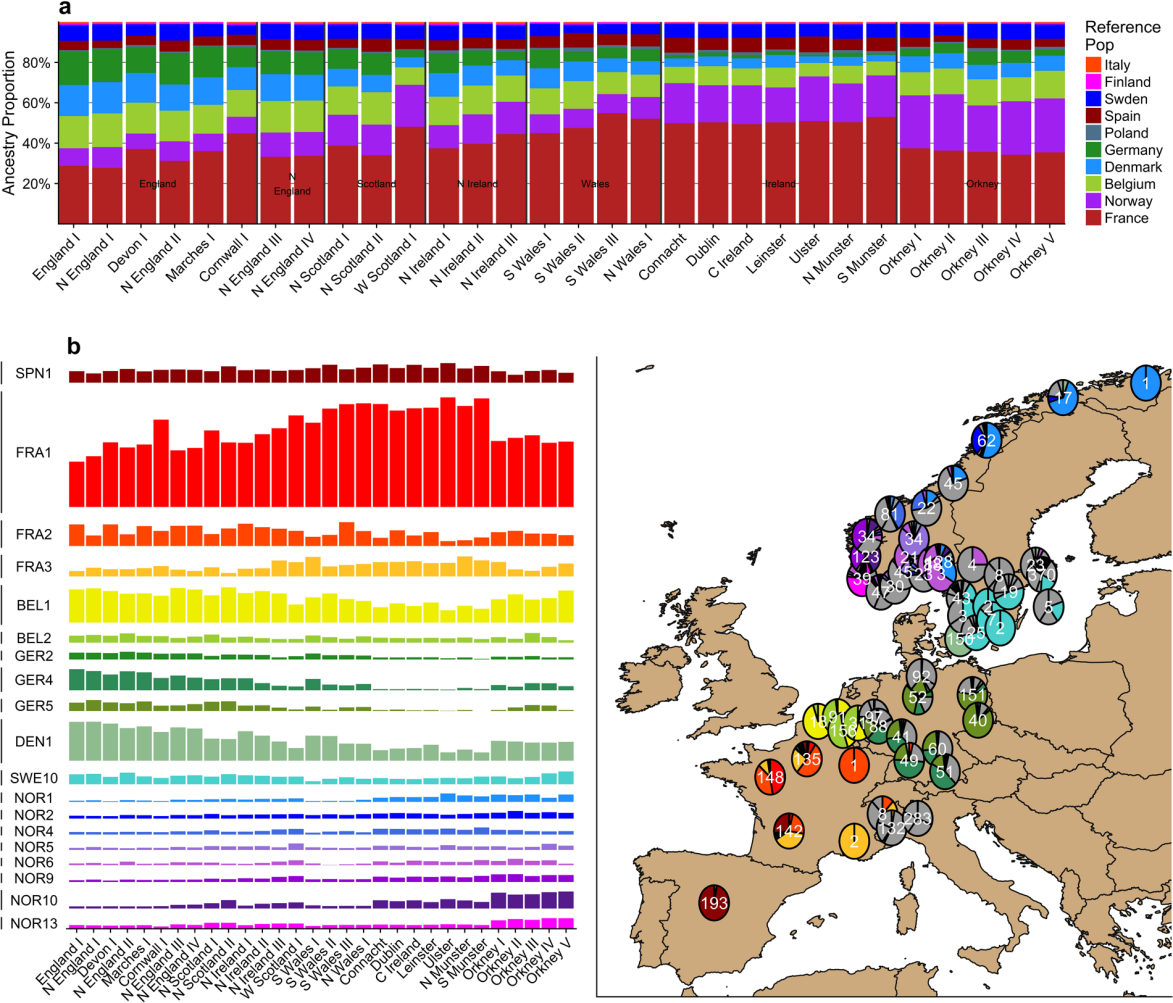
The European ancestry profiles of 30 Irish and British clusters. (**a**) The total ancestry contribution summarised by majority European country of origin to each of the 30 Irish and British clusters. (**b**) (left) The ancestry contributions of 19 European clusters that donate at least 2.5% ancestry to any one Irish or British cluster. (right) The geographic distribution of the 19 European clusters, shown as the proportion of individuals in each European region belonging to each of the 19 European clusters. The proportion of individuals form each European region not a member of the 19 European clusters is shown in grey. Total numbers of individuals from each region are shown in white text. Not all Europeans included in the analysis were phenotyped geographically. The figure was generated in the statistical software language R^46^, version 3.4.1, using various packages. The map of Europe was sourced from the R software package “mapdata” (https://CRAN.R-project.org/package=mapdata).

