# Supplementary material for: The Irish DNA Atlas: Revealing Fine-Scale Population Structure and History within Ireland
Source: Sci Rep. 2017 Dec 8;7:17199. doi: 10.1038/s41598-017-17124-4 (PMC5722868; doi:10.1038/s41598-017-17124-4)
Supplement: Supplementary file 1 — Supplemental Material [file 41598_2017_17124_MOESM1_ESM.pdf]

Supplemental Materials

Title

The Irish DNA Atlas: Revealing Fine Scale Population Structure and History within Ireland

Authors

Edmund Gilbert[1], Seamus O'Reilly[2], Michael Merrigan[2], Darren McGettigan[2], Anne M Molloy[3], Lawrence C Brody[4], Walter Bodmer[5], Katarzyna Hutnik[5], Sean Ennis[6], Daniel J Lawson[7], James F Wilson[8,9], Gianpiero L Cavalleri[1*]

Affiliations

[1]RCSI Molecular & Cellular Therapeutics, Royal College of Surgeons in Ireland, Dublin 2, Ireland. [2]Genealogical Society of Ireland, Dún Laoghaire, County Dublin, Ireland. [3]School of Medicine, Trinity College, Dublin 2, Ireland. [4]Genome Technology Branch, National Human Genome Research Institute, National Institutes of Health, Bethesda, MD 20892. [5]Weatherall Institute of Molecular Medicine and Department of Oncology, University of Oxford, Oxford OX3 7DQ, UK. [6]School of Medicine and Medical Science, University College Dublin, Dublin, Ireland. [7]University of Bristol, Department of Mathematics, University Walk, Bristol BS8 1TW, UK. [8]Centre for Global Health Research, Usher Institute for Population Health Sciences and Informatics, University of Edinburgh, Teviot Place, Edinburgh, Scotland. [9]MRC Human Genetics Unit, Institute of Genetics and Molecular Medicine, University of Edinburgh, Western General Hospital, Crewe Road, Edinburgh, Scotland.

Contents Page

Contents

Supplemental Materials ... 1
Title ... 1
Authors ... 1
Affiliations ... 1
Contents Page ... 2
Supplemental Data 1 – Study Populations ... 3
Supplemental Data 2 – Supplemental Methods ... 4
Supplemental Data 2.1 – The fineStructure Population Structure analysis. ... 4
Supplemental Data 2.2 – EEMS Analysis Pipeline ... 4
Supplemental Data 2.3 – The Ancestry Regression Method ... 5
Supplemental Data 3 – The Final Inferred Clustering of the Irish and British Datasets ... 6
Supplementary Figure 1 – The Final Inferred fineStructure Clustering within Ireland ... 6
Supplementary Table 1 – Ireland Britain fineStructure Clustering Details ... 7
Supplementary Figure 2 – Principal Component Analysis of the fineStructure Co-Ancestry Matrix ... 8
Supplemental Data 4 – Comparison of fineStructure PCA to comparable methods ... 9
Supplementary Figure 3 – Comparison of fineStructure PCA and GCTA64 PCA ... 10
Supplemental Data 5 – Comparison to Ancient Irish Genomes ... 11
Supplementary Table 3 – The overlap of common SNPs between two published ancient Irish individuals and a dataset of Irish and British individuals ... 11
Supplementary Figure 4 – Haplotypic affinity of Irish and British clusters to two ancient Irish genomes ... 12
Supplementary Figure 5 – Estimated Effective Migration Surface Diagnostic Plots ... 13
Supplemental Data 6 – Checking Ancestry Proportions ... 14
Supplementary Figure 6 – Ancestry profiles of 12 Norwegian populations ... 14
Supplementary Data 7 – GLOBETROTTER Joint Probability Curves ... 15
Supplementary Figure 7.1 – Joint Probability Curves of Irish clusters comparing Irish signal to primary Norwegian signal ... 16
Supplementary Figure 7.2 – Joint Probability Curves of *N Ireland I* ... 17
Supplementary Figure 7.3 – Joint Probability Curves of *N Ireland II* ... 18
Supplementary Figure 7.4 – Joint Probability Curves of *N Ireland III* ... 19
References ... 21

Supplemental Data 1 – Study Populations

Irish DNA Atlas

The Irish DNA Atlas (henceforth the Atlas) is a DNA cohort of individuals with Irish ancestry. To be included into the study, a participant must have all eight great-grandparents born within 50km, in Ireland. Therefore each Atlas individual is a sample of the genetics of specific regions in Ireland three generations ago. The Atlas is a collaborative project between the Royal College of Surgeons in Ireland, and the Genealogical Society of Ireland, and recruitment was primarily based through genealogical interest groups. Saliva samples were collected using Oragene OG-250 (DNAGenotek, Canada) collection devices, and DNA extracted according to standard protocol. Additionally each participant provided date of birth, and birth place information for all eight grandparents. Samples were then genotyped on the Illumina OmniExpress chip at Edinburgh Genomics, according to manufacturer's instructions. Informed consent was obtained from all individuals, and the data collection and analysis of these individuals was carried out in accordance with the relevant guidelines and regulations was approved by the Royal College of Surgeons in Ireland Research Committee, reference number REC0020563.

Trinity Student Study Cohort

This cohort consisted of 2232 students with Irish genetic ancestry recruited from Trinity College Dublin [1]. The sample was included as additional samples of the Irish population. Genotype information was generated using the Illumina 1M HumanOmni1-Quad chip.

Peoples of the British Isles Cohort

This cohort consisted of British individuals from the People of the British Isles (POBI) Study[2]. Individuals are phenotyped as having ancestry from 35 geographic regions across the United Kingdom. Genotype information from Illumina 1.2M platform was accessed via EBI, accession number EGAD00010000632.

WTCCC2 Project Multiple Sclerosis Cohort

In order to provide European haplotypes, we included European individuals from the WTCCC2 Multiple Sclerosis (MS) Study[3]. Genotype information from Illumina Human660-Quad chip platform was accessed via the European Genome and Phenome Archive, accession number EGAD00000000120.

Supplemental Data 2 – Supplemental Methods

Supplemental Data 2.1 – The fineStructure Population Structure analysis.

Using the final combined dataset of 2,103 individuals and 256,379 common markers we phased the dataset using SHAPEIT v2.r790[4] using an effective population size of 11,418 as suggested for European populations by the authors. We converted the resultant haplotype files to ChromoPainter format using the “impute2chromopainter2.pl” script (downloaded at http://www.paintmychromosomes.com/). For the phasing and conversion we used genetic map build 37 downloaded with SHAPEIT.

Population structure analysis was performed by the combined software fineStructure (a pre-release version, 2.1.0.pre)[5], which includes the software ChromoPainter, Chromocombine, and fineStructure. Chromopainter was applied using default settings, with the exception of specifying the number of ‘chunks’ per region to 50 as other analyses[10] have found that British and Irish individuals share relatively longer haplotypes than average. We ‘painted’ each individual using every other individual in the analysis as a donor using the -a 0 0 switch. Principal component analysis (PCA) was performed on the resultant co-ancestry matrix. We then performed fineStructure clustering MCMC analysis on the resultant co-ancestry matrix; with 1,000,000 burnin iterations, 1,000,000 sampling iterations, and retaining 500 MCMC samples. With the MCMC sample with the highest posterior probability we performed 1,000,000 additional hill climbing moves to reach the final inferred clustering and tree. When tree building we utilised the -T 1 parameter within fs-2.1.0.pre, which uses the Maximum Concordance State method first reported by Leslie et al[6].

Supplemental Data 2.2 – EEMS Analysis Pipeline

Atlas individual latitude and longitude coordinates were were generated from the average of their eight great-grandparents’ birth places. The coordinates for the habitat boundaries were generated with an online Google Maps API tool (http://www.birdtheme.org/useful/v3tool.html), and the matrix of average pair-wise genetic dissimilarities was generated from plink format data using the bed2diffs software included in the EEMS download package.

At the beginning of the analytical pipeline ten independent MCMC chains were started, each with a random random-number-seed, for an initial 100,000 burnin and 100,000 sampling iterations (thinning every 999 iterations), placing samples to the nearest of 600 demes. We chose the chain with the highest final log-likelihood, and started 10 new EEMS chains, using this chain as a starting point. This second round of chains were each started with a random random-number-seed, with 1,000,000 burnin iterations and 1,000,000 sampling iterations, sampling every 9,999 iteration – placing samples to the nearest of 800 demes. We removed two chains (8 and 10) as these has

consistently lower log-likelihoods. We then checked whether their exclusion significantly changed the predictive power of the EEMS model by noting the change in the r^2 value of the expected versus fitted dissimilarities between demes (0.326 verses 0.325 without). We plotted the results of our EEMS analysis using the R[7] package, “rEEMSplots”, which is included in the EEMS software download. We used all final eight EEMS runs as input, and plotted the average estimated migration and diversity surfaces, the posterior probability trace log for all eight chains, and a scatter plot of observed vs fitted genetic dissimilarities within demes. The dissimilarity between observed versus fitted deme pairs show a general trend with some deviation (r^2 = 0.325) (Supplementary Figure 5a) and the log-posterior trace of the eight replicate MCMC chains (Supplementary Figure 5b) show convergence of the independent EEMS runs.

Supplemental Data 2.3 – The Ancestry Regression Method

To investigate the genetic ancestry of any Irish clusters we observe we utilised a regression based “ancestry profile” method first described by Leslie et al[6]. Briefly we estimated the proportion of ancestry in each Irish and British individual that most closely resembles that represented by different European, reference, individuals. These proportions can then be summed across groups. We considered Y_P which is a vector of *G* length (where *G* is the number of European reference populations), recording the average length of DNA genome-wide that each *G*-population donates to Irish or British cluster *P* – which is then summed to unity across the vector. Additionally, X_G is a vector of *G*-length that records the average length of DNA genome-wide that each European individual copies from each European *G* population (with individuals unable to copy their own haplotype to themselves). With,

$$Y_P = \beta_1 X_1 + \beta_2 X_2 + \ldots + \beta_G X_{G,}$$

we solve β_g assuming $\beta_g \geq 0$ and $\sum_{g=1}^{G} \beta g = 1$, using an adaptation of the non-negative-least-squares (nnls) function in R[7, 8]. Each inferred value of β_g is interpreted as the average proportion of ancestry of genome-wide DNA each Irish or British individual from cluster *P* that is most closely related, ancestrally, to each European cluster *g*.

To calculate confidence intervals we performed bootstrapping, resampling the chromosomes of the Irish and British individuals and creating pseudo-individuals from the sampled chromosomes. We recalculated our estimates of β_g from these pseudo-individuals to compute the 95% confidence intervals over 1000 bootstrap intervals.

Supplemental Data 3 – The Final Inferred Clustering of the Irish and British Datasets

The final inferred state of fineStructure clusters in our analysis of Irish and British individuals (see Methods and Supplemental Data 2.1) yielded a total of 48 clusters, with many of these clusters numbers <10 individuals (n = 16). We investigated the clustering at each hierarchal value of *k*-clusters, paying attention to the cluster sizes and the degree of Irish substructure shown. We found that at *k* = 30, the 7 major clusters of predominantly Irish membership are separated (with two clusters of two Irish individuals each merged with the *Connacht* and the *N Munster* clusters respectively).

We show the geographic distribution of Atlas samples according to cluster membership (Figure S1A), the final inferred fineStructure tree at *k* = 48 (Figure S1B), and the membership of each *k* = 48 cluster in each *k* = 30 cluster with cluster size shown (Table S1).

Supplementary Figure 1 – The Final Inferred fineStructure Clustering within Ireland

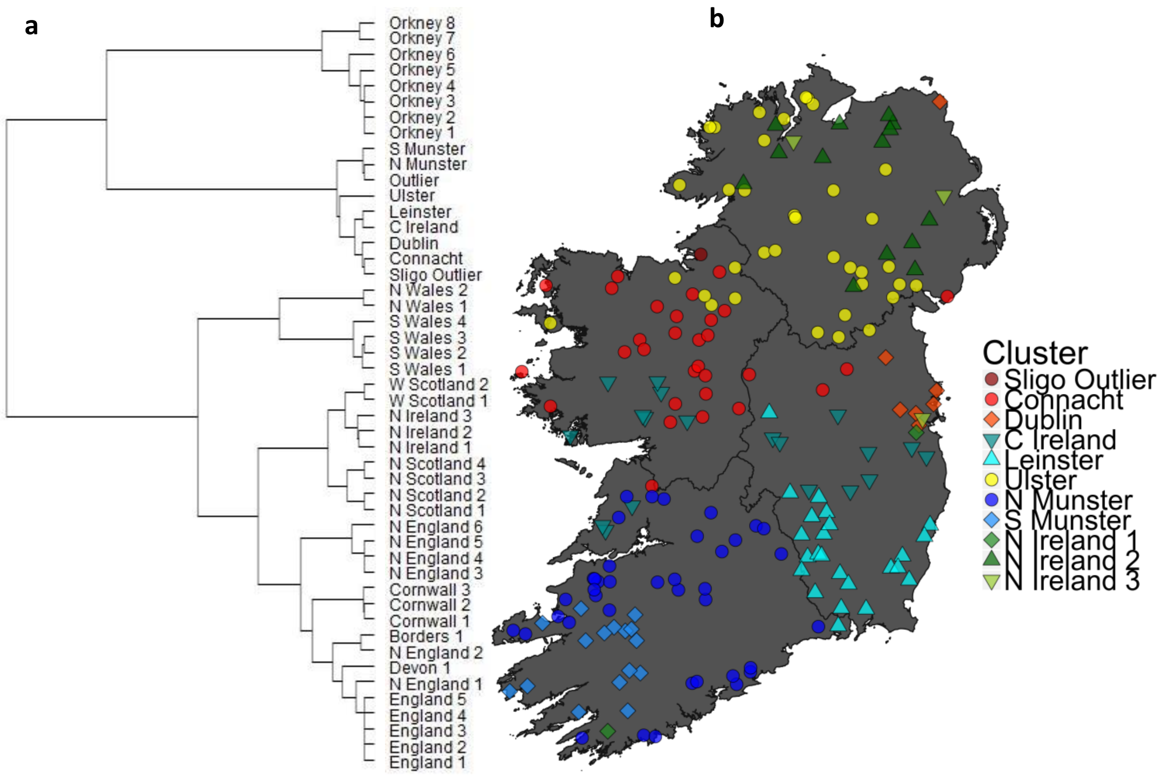

Supplementary Figure 1 - The final inferred fineStructure clustering state of 2,103 Irish and British individuals. (a) The fineStructure dendrogram of the 48 final inferred clusters. (b) The geographic spread of the clusters containing Atlas Irish individuals, colour and shaped coded according to fineStructure cluster membership. Geographic location is the average of the Atlas individuals' great-grandparental birth places. Open Street Map Ireland, Copyright OpenStreetMap Contributors, (https://www.openstreetmap.ie/) - data available under the Open Database Licence. The figure was plotted in the statistical software language R, version 3.4.1[7], with various packages.

Supplementary Table 1 – Ireland Britain fineStructure Clustering Details

The individual final inferred 48 clusters and which K30 cluster that cluster is a member of. Also shown are the individual sizes for each K48 and K30 cluster.

K48 Cluster	K48 Cluster Size	K30 Cluster	K30 Cluster Size
England 1	1	England I	
England 2	1	England I	
England 3	528	England I	536
England 4	3	England I	
England 5	3	England I	
N England 1	82	N England I	82
Devon 1	73	Devon I	73
N England 2	32	N England II	32
Marches 1	78	Marches I	78
Cornwall 1	5	Cornwall I	
Cornwall 2	13	Cornwall I	82
Cornwall 3	64	Cornwall I	
N England 3	2	N England III	
N England 4	149	N England III	153
N England 5	2	N England III	
N England 6	149	N England IV	149
N Scotland 1	6	N Scotland I	39
N Scotland 2	33	N Scotland I	
N Scotland 3	7	N Scotland II	14
N Scotland 4	7	N Scotland II	
N Ireland 1	33	N Ireland I	33
N Ireland 2	94	N Ireland II	94
N Ireland 3	38	N Ireland III	38
W Scotland 1	7	W Scotland I	23
W Scotland 2	16	W Scotland I	23
S Wales 1	11	S Wales I	11
S Wales 2	3	S Wales II	26
S Wales 3	23	S Wales II	
S Wales 4	22	S Wales III	22
N Wales 1	63	N Wales I	75
N Wales 2	12	N Wales I	75
Sligo Outlier	2	Connacht	96
Connacht	94	Connacht	
Dublin	48	Dublin	48
C Ireland	77	C Ireland	77
Leinster	62	Leinster	62
Ulster	61	Ulster	61
Outlier	2	N Munster	76
N Munster	74	N Munster	
S Munster	28	S Munster	28
Orkney 1	2	Orkney I	
Orkney 2	12	Orkney I	38
Orkney 3	4	Orkney I	
Orkney 4	20	Orkney I	
Orkney 5	13	Orkney II	13
Orkney 6	15	Orkney III	15
Orkney 7	13	Orkney IV	13
Orkney 8	16	Orkney V	16

Supplementary Figure 2 – Principal Component Analysis of the fineStructure Co-Ancestry Matrix

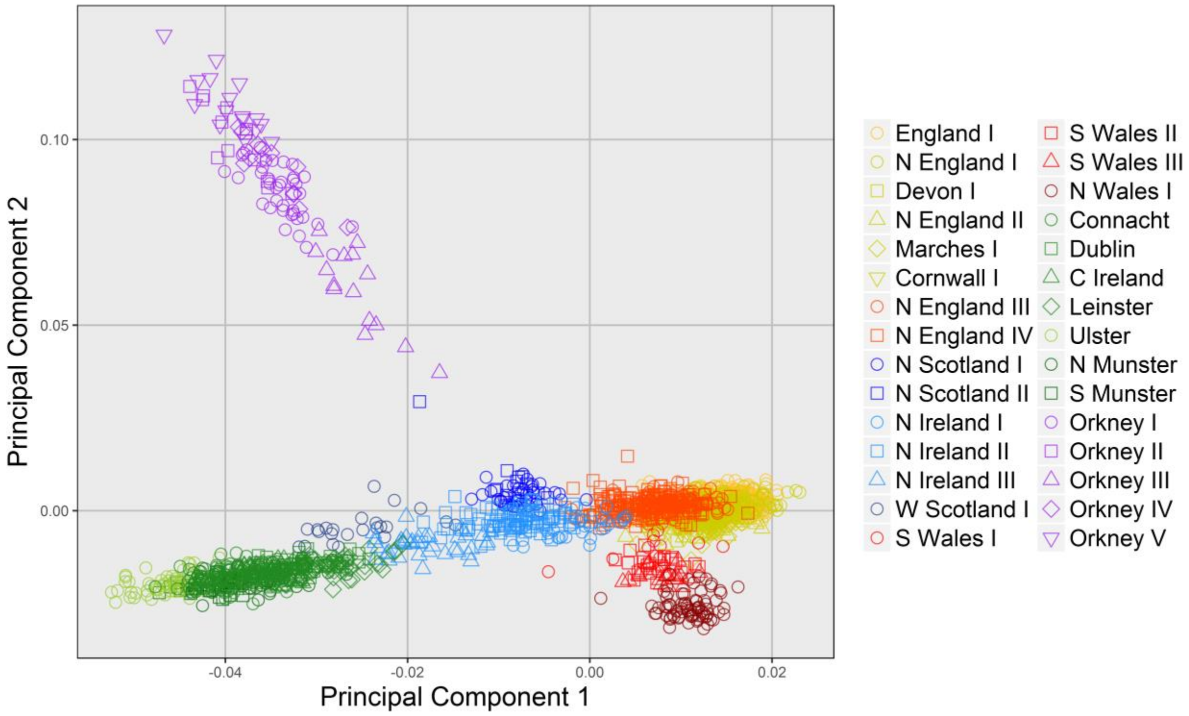

Supplementary Figure 2 - The Principal component analysis of the fineStructure co-ancestry matrix. Shown are the first and second principal components, with individuals labelled according to k = 30 fineStructure cluster membership.

Supplemental Data 4 – Comparison of fineStructure PCA to comparable methods

To compare fineStructure's ability to differentiate population structure to more conventional methods we performed principal component analysis (PCA) of the Irish and British dataset used in our analysis of Population Structure within Ireland using gcta64[9], and compared this to the PCA of the fineStructure co-ancestry matrix. We generated principal components from the co-ancestry matrix using methods previously described in Supplemental Data 2.1. In order to generate the principal components using gcta64, we used plink 1.9[10, 11] formatted data of the 2,103 Irish and British individuals and the 256,379 common markers. We first pruned the dataset of SNPs with the plink command --indep-pairwise 1000 50 0.2. Then, using a pruned dataset of 79,417 common markers, we generated a genetic relationship matrix (grm) with gcta64, and finally generated the top 10 principal components from this matrix using gcta64's "--pca" function.

As previously reported[5, 6], the haplotype-based fineStructure shows a greater ability to differentiate population structure than more conventional allele frequency based methods such as gcta64. This is demonstrated at the higher components as well as lower principal components (shown are principal components 7 and 8 in Figure S3). fineStructure's ability to differentiate population structure at lower components (where gcta64 is not able to detect structure) presumably reflective of its ability to detect the fine scale structure that we observe within Ireland.

Supplementary Figure 3 – Comparison of fineStructure PCA and GCTA64 PCA

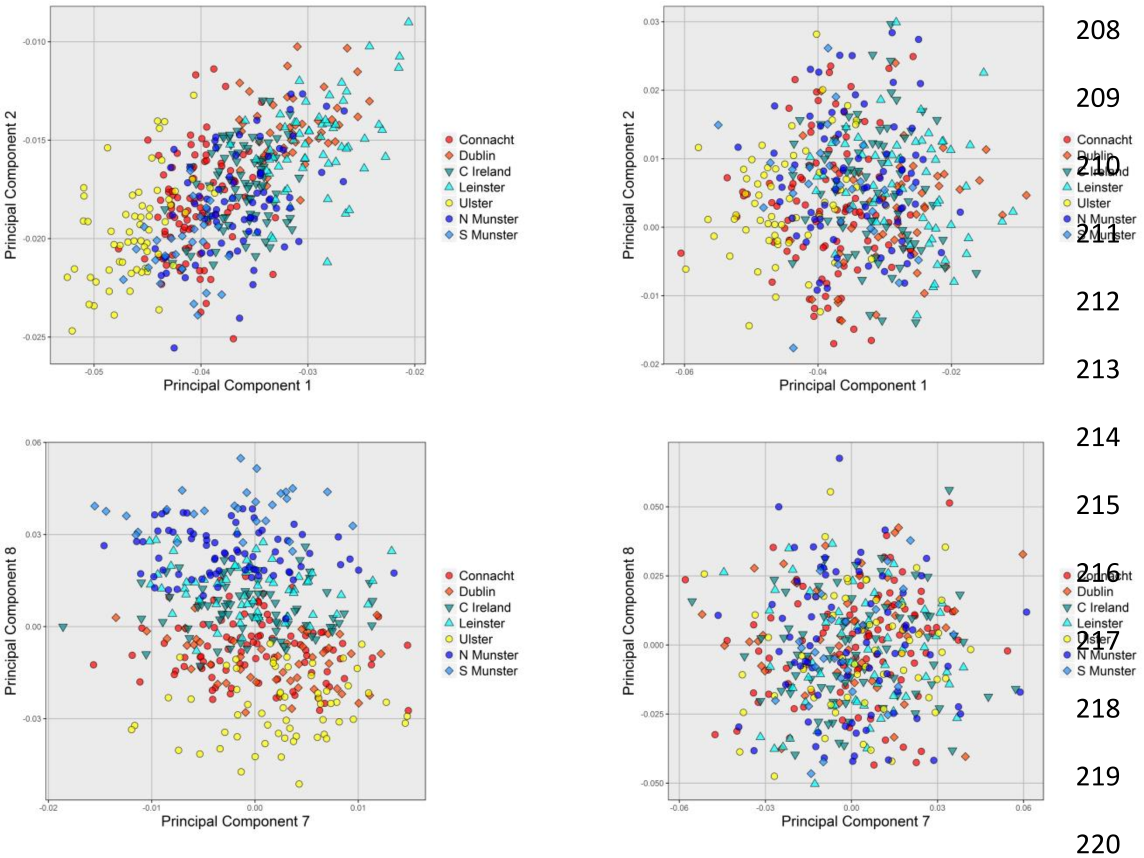

Supplementary Figure 3 – Comparison of fineStructure-based principal component analysis (PCA) and conventional PCA methods. Shown are all individuals included in the Irish and British fineStructure analysis that were placed in clusters on the Irish branch at *k* = 30. We show, with four panels, their PCA coordinates along PC principal components 1 and 2 (upper two) and principal components 7 and 8 (lower two) using fineStructure (left two) and gcta64 (right two).

Supplemental Data 5 – Comparison to Ancient Irish Genomes

We decided to compare the Atlas Irish individuals in our sample to two previously published[12] high coverage ancient Irish genomes; a Neolithic farmer (Ballynahatty) and a Bronze Age individual (Rathlin1). The authors of the aforementioned authors found the greatest affinity to the modern Irish was found in the Bronze Age individual studied. We set out to investigate whether any particular region in Ireland as represented in our Atlas Irish individuals and Irish fineStructure clusters shared an affinity to either of the ancient Irish individuals.

We found the intersect of common shared SNPs between the individuals included in the fineStructure analysis of Population Structure within Ireland (see methods for more detail) individually for each ancient Irish individual (see Table S3 for SNP overlaps).

Supplementary Table 3 – The overlap of common SNPs between two published ancient Irish individuals and a dataset of Irish and British individuals

Ancient Irish	Ballynahatty	Rathlin1
SNP Overlap	162,069	175,749

We merged the Irish and British dataset individually with each ancient individual, and as each ancient individuals was of high coverage (>10x), we phased and performed fineStructure analysis separately. We phased each of the datasets using SHAPEIT[4], using the same method outlined by Cassidy et al[12]. We painted each individual donating haplotypes to every other individual in the analysis (the "-a" switch). We report the average haplotypic donation from each ancient Irish genome to each modern Irish or British cluster in cM. We calculated the standard error for each Irish or British cluster from the standard error of the individual sample lengths within each cluster.

Supplementary Figure 4 – Haplotypic affinity of Irish and British clusters to two ancient Irish genomes

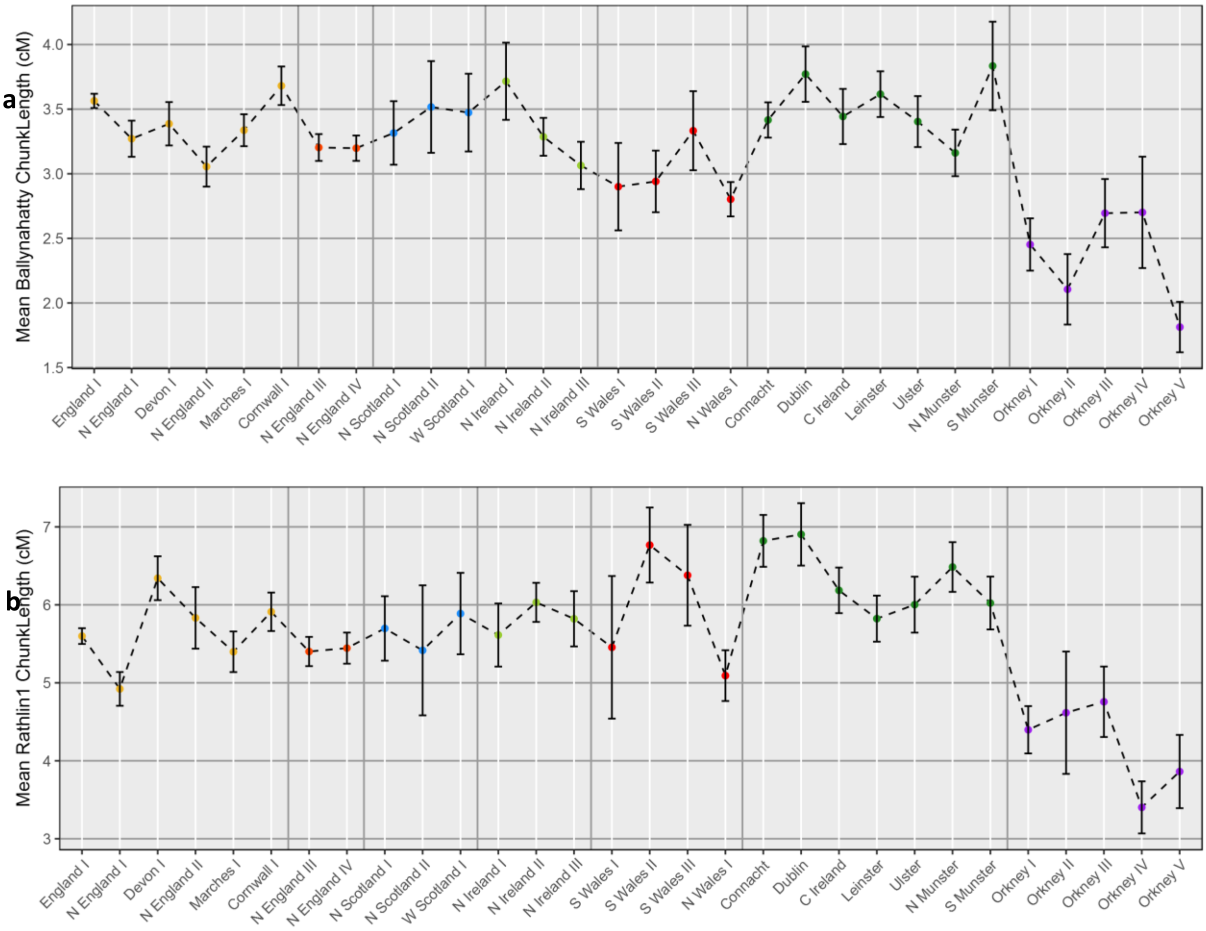

Supplementary Figure 4 – Haplotypic Affinity of each Ancient Irish genome to each modern Irish and British cluster. Shown is the average length of haplotype donation from each ancient individual, Ballynahatty (a) and Rathlin (b), to each k = 30 Irish and British cluster in cM. Also shown are the standard error bars calculated from the standard error of the sample lengths of the individuals within each of the individual clusters.

Supplementary Figure 5 – Estimated Effective Migration Surface Diagnostic Plots

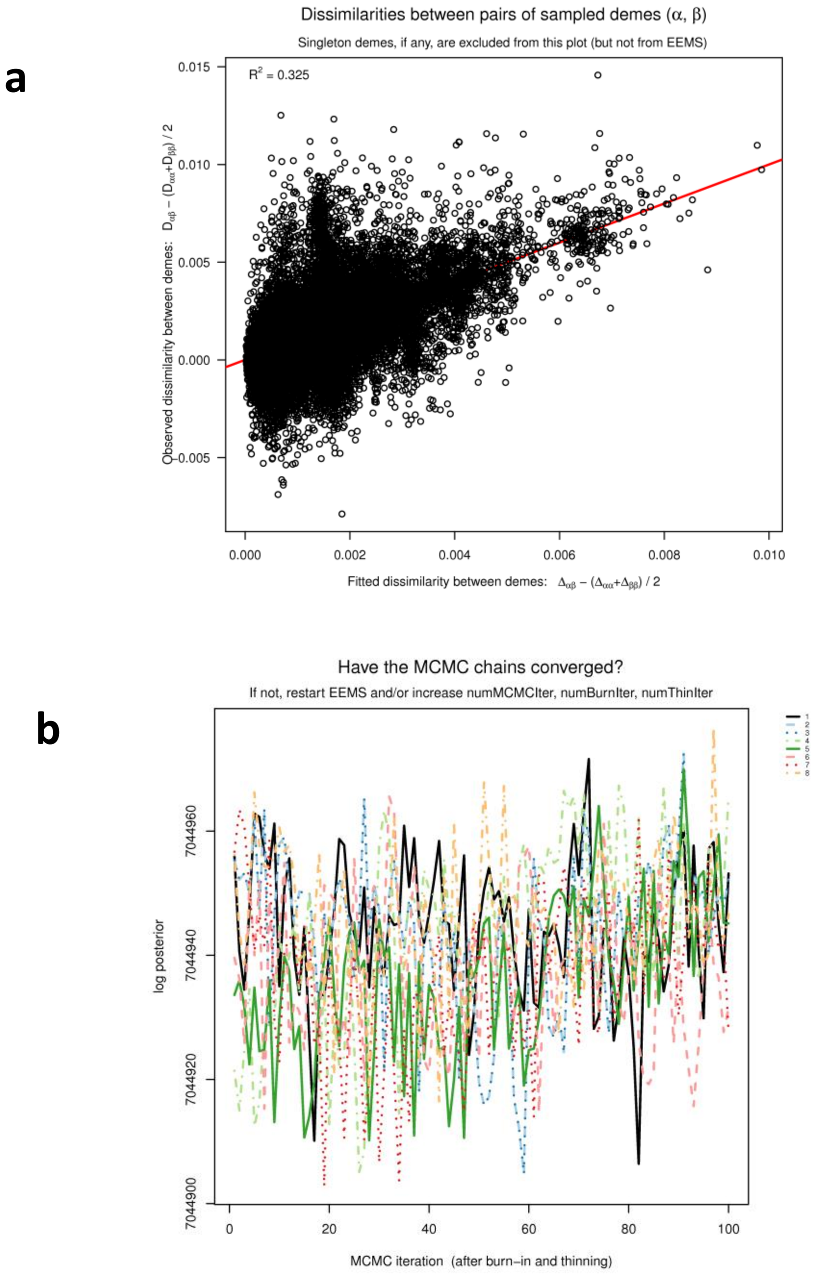

Supplementary Figure 5 – Estimated Effective Migration Surface Diagnostic plots. (a) The observed versus expect dissimilarity between pairs of demes (with demes with only one individual not considered). Strong deviations from the fitted line (red) indicate pairs of demes much more genetically distant than expected. (b) The posterior probability log of the six replicates of the EEMS run, indicating whether the MCMC chains have converged.

Supplemental Data 6 – Checking Ancestry Proportions

Our regression based ancestry profile analysis of Irish and British haplotypes revealed a surprising amount of Norwegian-like ancestry in our Irish samples. To investigate whether this was due wholly or in part to Irish haplotypes existing in our modern Norwegian sample we performed an additional regression based ancestry analysis. We modelled the Norwegian k = 51 clusters as a mixture of the other k = 51 European clusters, as well as the k = 30 Irish and British clusters using the same methodology as described in the Materials and Methods, as well as Supplementary Data 2.1. We present the data as the average reference country contribution to each Norwegian cluster, where Norwegian clusters are organised by region of origin within Norway.

Supplementary Figure 6 – Ancestry profiles of 12 Norwegian populations

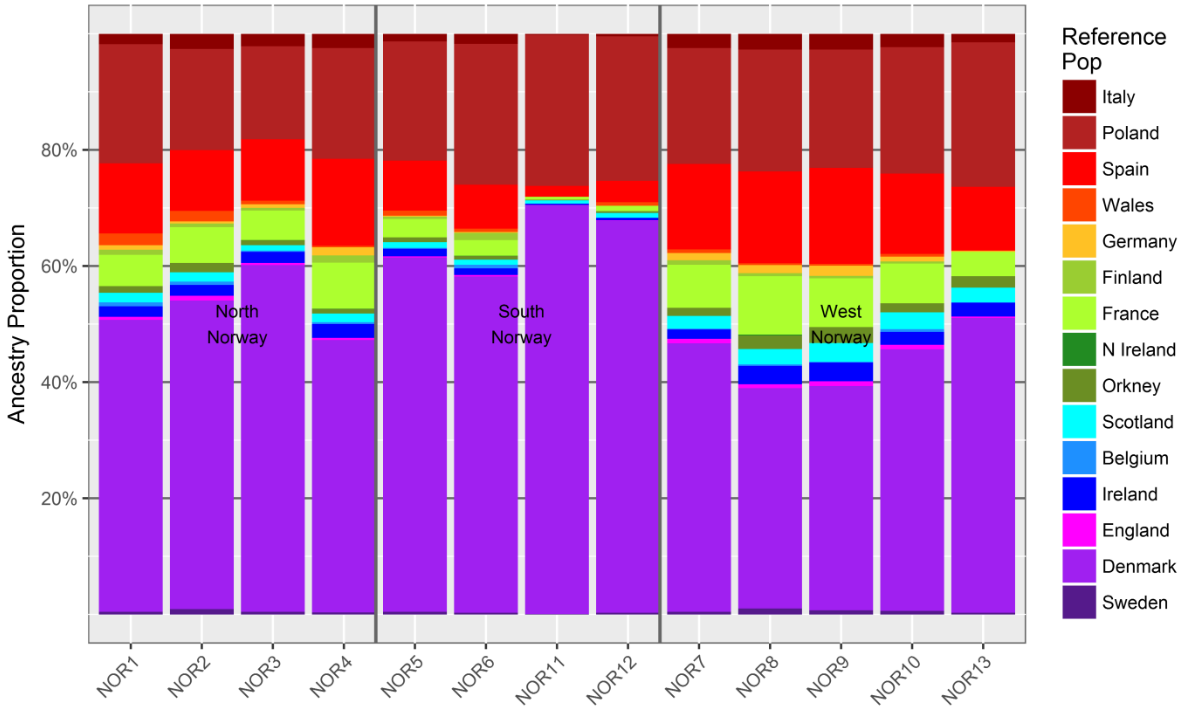

Supplementary Figure 6 – Ancestry profiles of 13 Norwegian populations modelled as a mixture of Irish and British, and mainland European populations. Shown are the average total ancestry contributions of all reference fineStructure clusters where the majority of individuals originate from each country, in each Norwegian cluster. Norwegian clusters are organised in three groups based on where the majority of individuals are from in Norway in each cluster.

Supplementary Data 7 – GLOBETROTTER Joint Probability Curves

To investigate the evidence of admixture events within Ireland we performed Globetrotter[13] analysis on a number of different fineStructure identified clusters of Irish membership. Firstly, we investigated the evidence of European admixture into Ireland. We present the joint probability plot for each Irish cluster, showing the primary Norwegian cluster versus the main component on the putative Gaelic source Figure S7.1. Secondly we investigated evidence that the three clusters of joint Irish and British membership were the result of admixture event(s) between Irish and British clusters, the results of which are shown in Table S8. We also present the joint probability curves for the major surrogate components for each source population, or each *N Ireland* cluster analysis (see below, Figures S7.2-4).

Each joint probability curve shows the probability that two positions, separated by genetic distance *x*, corresponds to ancestry donated by population A and population B, where populations A and B can be the same population. These can be used to assess the strength of the admixture signal detected by Globetrotter, the cleaner the signal around the fitted curve and the steeper the curve, the stronger the admixture signal. When population A=A or B=B and the fitted curve is negative, and the fitted curve when A as compared to B is positive it is indicative of admixture.

Supplementary Figure 7.1 – Joint Probability Curves of Irish clusters comparing Irish signal to primary Norwegian signal

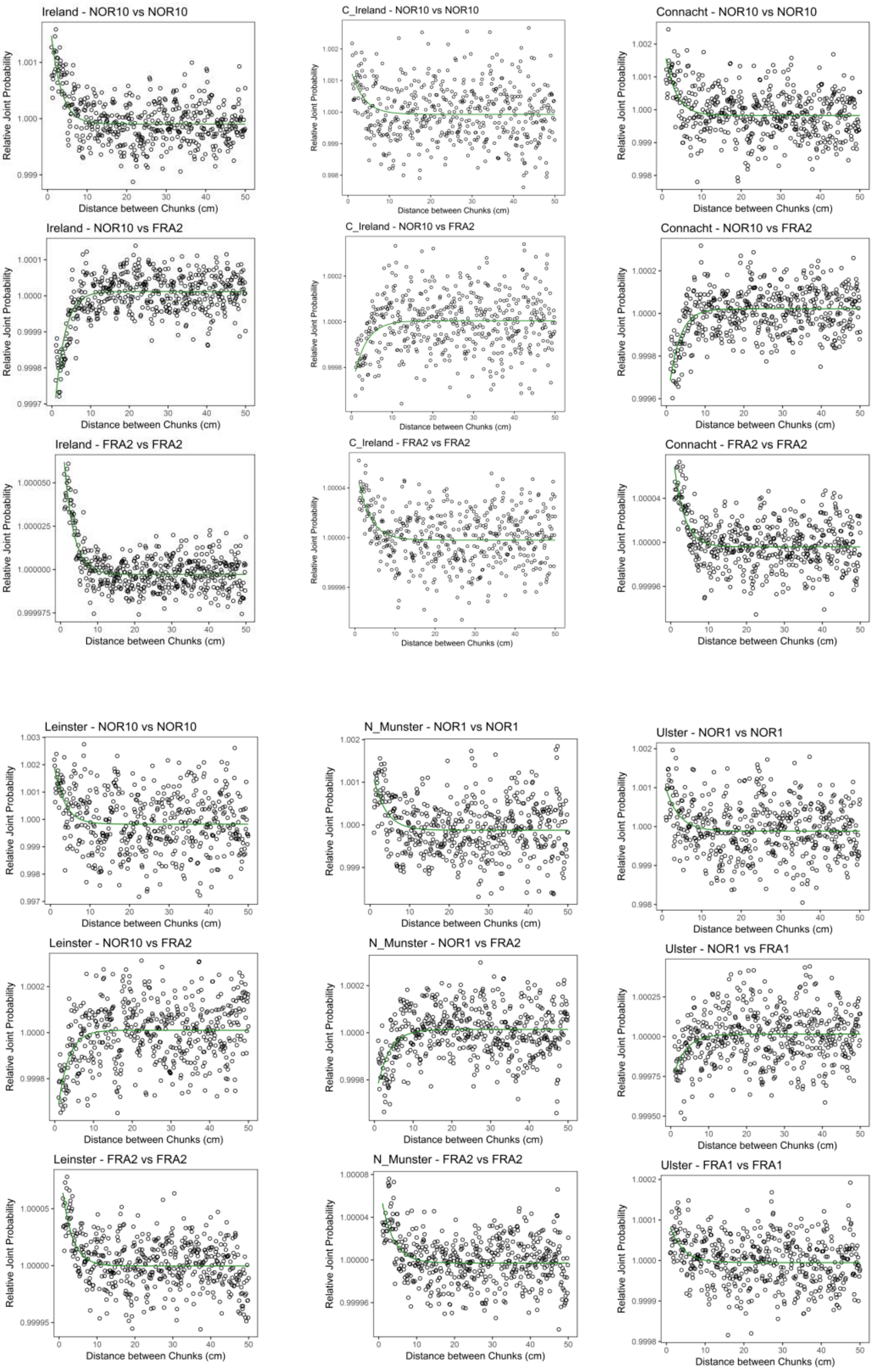

Supplementary Figure 7.1 – The admixture probability curves of the major components of the Gaelic Irish admixture events. The joint probability curves of the 6 Gaelic Irish clusters that show evidence of a significant admixture signal; (top left to bottom right), *Ireland*, *C Ireland*, *Connacht*, *Leinster*, *N Munster*, and *Ulster*. The scaled probability data is shown in black, with the fitted curve shown in green.

Supplementary Figure 7.2 – Joint Probability Curves of *N Ireland I*

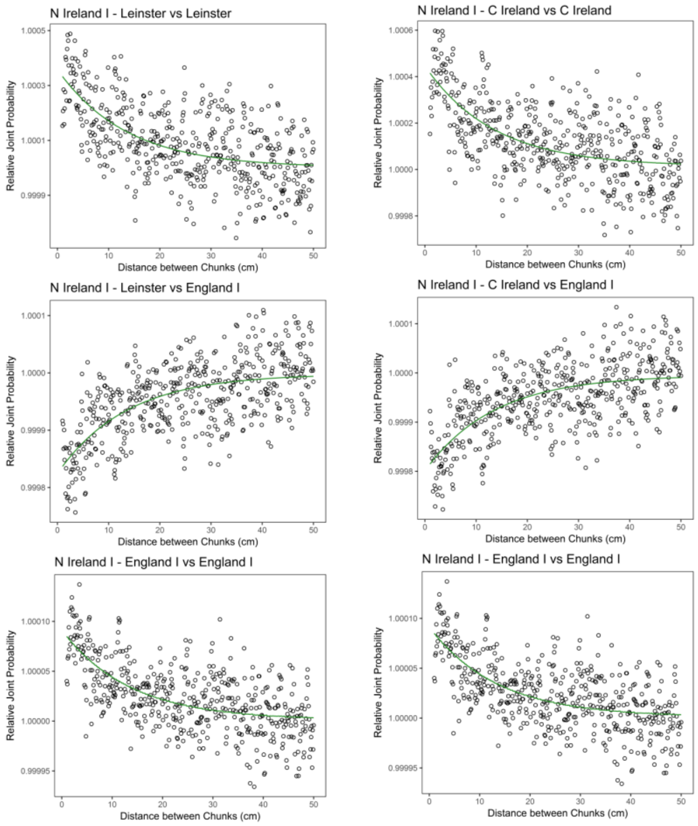

Supplementary Figure 7.2 – The admixture probability curves of the major components of the *N Ireland I* admixture event. (left) The three sets of probability curves showing the primary British (*England I*) and Irish (*Leinster*) components of the admixture event, and the probability that two sites separated by genetic distance *x* are donated from population A and B. (right) The same as the left panels, with the secondary Irish component, *C Ireland*. For both the scaled probability data is shown in black, with the fitted curve shown in green.

Supplementary Figure 7.3 – Joint Probability Curves of *N Ireland II*

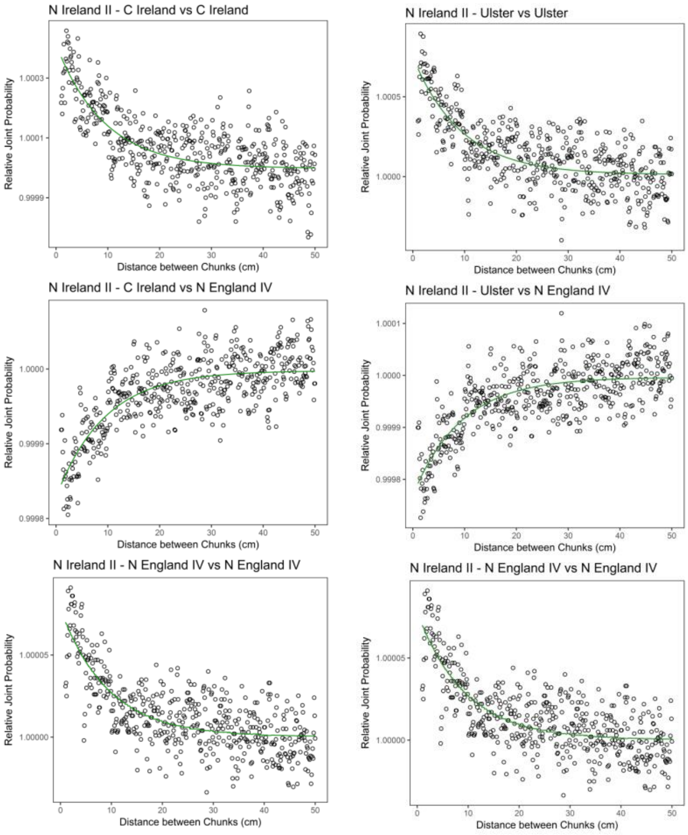

Supplementary Figure 7.3 – The admixture probability curves of the major components of the *N Ireland II* admixture event. (left) The three sets of probability curves showing the primary British (*N England IV*) and Irish (*Leinster*) components of the admixture event, and the probability that two sites separated by genetic distance *x* are donated from population A and B. (right) The same as the left panels, with the secondary Irish component, *C Ireland*. For both the scaled probability data is shown in black, with the fitted curve shown in green.

Supplementary Figure 7.4 – Joint Probability Curves of *N Ireland III*

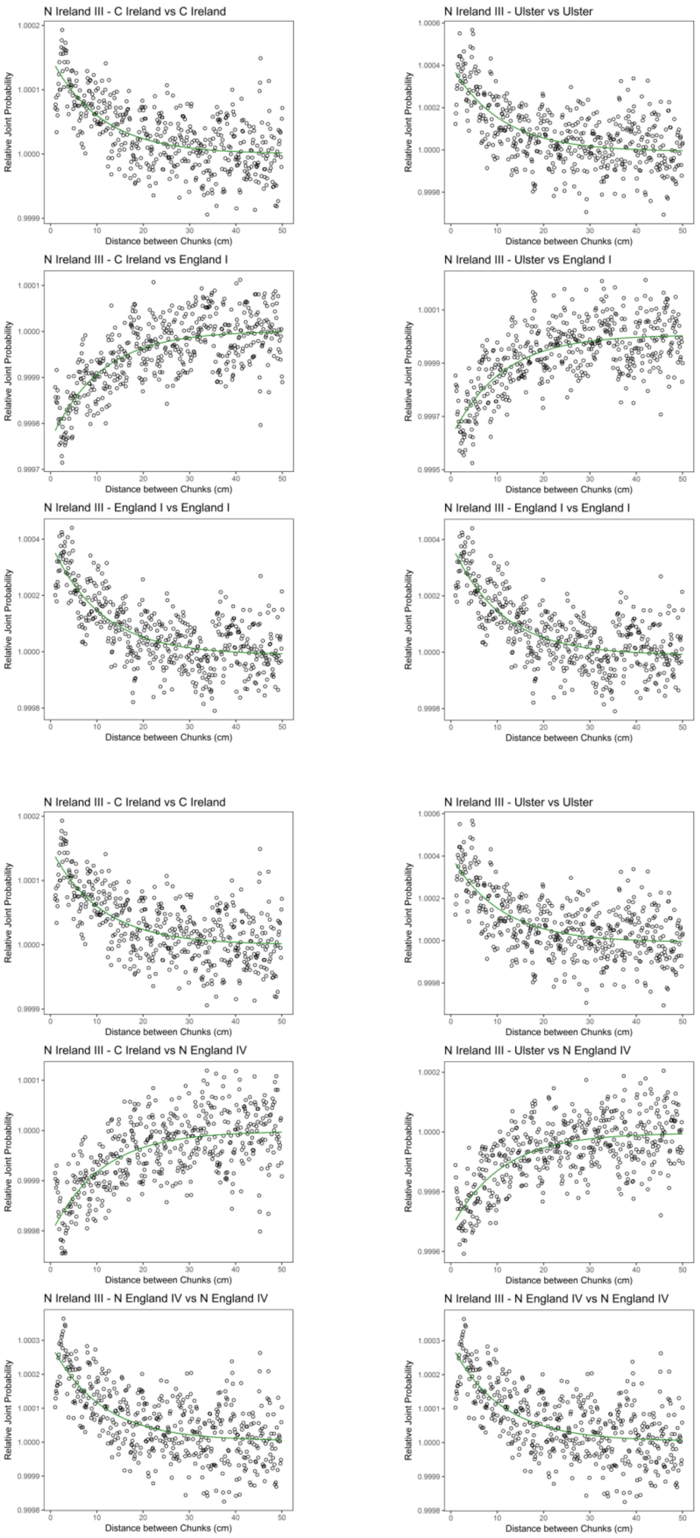

Supplementary Figure 7.4 – The admixture probability curves of the major components of the *N Ireland III* admixture event. (top-left) The three sets of probability curves showing the primary British (*England I*) and Irish (*C Ireland*) components of the admixture event, and the probability that two sites separated by genetic distance *x* are donated from population A and B. (top-right) The same

as the left panels, with the secondary Irish component, *Ulster*. (bottom-left) As top-left, with the secondary British component, *N England IV*, and the primary Irish component, *C Ireland*. (bottom-right) As top-left, but with the secondary British component, *N England IV*, and the secondary Irish component, *Ulster*. For both the scaled probability data is shown in black, with the fitted curve shown in green.

References

1. Desch KC, et al., *Linkage analysis identifies a locus for plasma von Willebrand factor undetected by genome-wide association.* Proc Natl Acad Sci USA, 2013. **110**(2): p. 588-93.
2. Winney B, et al., *People of the British Isles: preliminary analysis of genotypes and surnames in a UK-control population.* Eur J Hum Genet, 2012. **20**(2): p. 203-10.
3. IMSGC WTCCC2, *Genetic risk and a primary role for cell-mediated immune mechanisms in multiple sclerosis.* Nature, 2011. **476**(7359): p. 214-219.
4. Delaneau O, Marchini J, and Zagury JF, *A linear complexity phasing method for thousands of genomes.* Nature Methods, 2011. **9**(2): p. 179-81.
5. Lawson DJ, et al., *Inference of population structure using dense haplotype data.* PLoS Genet, 2012. **8**(1): p. e1002453.
6. Leslie S, et al., *The fine-scale population structure of the British population.* Nature, 2015. **519**: p. 309-14.
7. R Core Team, *R: A Language and Environment for Statistical Computing*. 2017, R Foundation for Statistical Computing: Vienna, Austria.
8. Lawson CL and Hanson RJ, *Solve Least Squares Problems*. 1995: Reprinted by the Society for Industrial and Applied Mathematics.
9. Yang J, et al., *GCTA: a tool for genome-wide complex trait analysis.* Am J Hum Genet, 2011. **88**(1): p. 76-82.
10. Purcell S, et al., *PLINK: a tool set for whole-genome association and population-based linkage analyses.* Am J Hum Genet, 2007. **81**(3): p. 559-75.
11. Chang CC, et al., *Second-generation PLINK: rising to the challenge of larger and richer datasets.* Gigascience, 2015. **4**: p. 7.
12. Cassidy LM, et al., *Neolithic and Bronze Age migration to Ireland and establishment of the insular Atlantic genome.* Proc Natl Acad Sci USA., 2016. **113**(2): p. 368-73.
13. Hellenthal G, et al., *A genetic atlas of human admixture history.* Science, 2014. **343**(6172): p. 747-51.
